# Dataset of Finite Element Models of Normal and Deformed Thoracolumbar Spine

**DOI:** 10.1038/s41597-024-03351-8

**Published:** 2024-05-29

**Authors:** Morteza Rasouligandomani, Alejandro del Arco, Francis Kiptengwer Chemorion, Marc-Antonio Bisotti, Fabio Galbusera, Jérôme Noailly, Miguel A. González Ballester

**Affiliations:** 1 BCN MedTech, Department of Engineering, Universitat Pompeu Fabra, Barcelona, 08018 Spain; 2 Hospital del Mar, Barcelona, 08003 Spain; 3 InSilicoTrials Technologies Company, Trieste, 34123 Italy; 4 Barcelona Supercomputing Center, Barcelona, 08034 Spain; 5 IRCCS Istituto Ortopedico Galeazzi, Milan, 20161 Italy; 6 ICREA, Barcelona, 08010 Spain; 7 Present Address: Schulthess Klinik, Zürich, 8008 Switzerland

**Keywords:** Computational models, Biomedical engineering, Mechanical engineering

## Abstract

Adult spine deformity (ASD) is prevalent and leads to a sagittal misalignment in the vertebral column. Computational methods, including Finite Element (FE) Models, have emerged as valuable tools for investigating the causes and treatment of ASD through biomechanical simulations. However, the process of generating personalised FE models is often complex and time-consuming. To address this challenge, we present a dataset of FE models with diverse spine morphologies that statistically represent real geometries from a cohort of patients. These models are generated using EOS images, which are utilized to reconstruct 3D surface spine models. Subsequently, a Statistical Shape Model (SSM) is constructed, enabling the adaptation of a FE hexahedral mesh template for both the bone and soft tissues of the spine through mesh morphing. The SSM deformation fields facilitate the personalization of the mean hexahedral FE model based on sagittal balance measurements. Ultimately, this new hexahedral SSM tool offers a means to generate a virtual cohort of 16807 thoracolumbar FE spine models, which are openly shared in a public repository.

## Background & Summary

The Finite Element (FE) Method is well-known for the numerical solving of differential equations through the discretization of complex geometrical domains into discrete cells, or elements, in which physics-based partial differential equations can solved. FE analyses have shown their value to explore causatively spine pathologies, or spine surgical treatments and devices^1–4^. Recently, several studies focused on FE simulations of lumbar spine deformity (only scoliosis), spine surgery, and mechanical response of Intervertebral Disk (IVD) and soft tissues. However, there was lack of biomechanical FE models for thoracolumbar spine sagittal deformities.

As spine sagittal deformity is a patient-specific deviation from the standard spine geometry, the modelling thereof needs to be personalised. Clinical images of the lumbar and thoracolumbar spine have been exploited in patient-specific FE modelling of the spine^5–8^. Furthermore, image analysis techniques such as statistical shape analysis have led to Statistical Shape Models (SSM), providing a powerful tool to describe morphological variations in a specific population and use this knowledge to personalize the models according to specific patient characteristics. SSM has been proposed for the lumbar, thoracic, and cervical spines^9–13^. Yet, spine sagittal deformity and morphological balance explorations require the modelling of, at least, the thoracolumbar spine including the pelvis and sacrum, and the femoral head^14^.

In biomechanical FE models, such as spine FE model, shapes need to be discretized, or meshed, into elements for the iterative solving of the partial differential equations that mathematically describe the physics of the mechanically loaded organ. The meshes can be classified in two main categories: triangulated and hexahedral. In contrast to triangulated meshes, hexahedral meshes follow the structural organization of the tissues that build the organ. They are particularly convenient to define the local anisotropic material properties or oriented material reinforcements, found in biological tissues^15^.

Hexahedral meshes are the most accurate FE meshes which allow optimal mesh convergence for the numerical solving of systems of partial differential equations. This becomes particularly evident under large deformations, e.g., as experienced by spine IVDs^16,17^. However, their automatic adaptation to nonlinear tissue and organ shapes is challenging and require cumbersome manual operations, especially when used in structural meshes. As automatic segmentations of medical images provide triangulated surface meshes, the creation of 3D structural meshes out of these segmentations stands for a strong limitation, therefore, to define numerically optimal patient-specific spine FE models. Recently, a few studies focused on the thoracolumbar spine FE hexahedral meshes, in which no sagittal deformity had been observed.

**Fig. 1:**
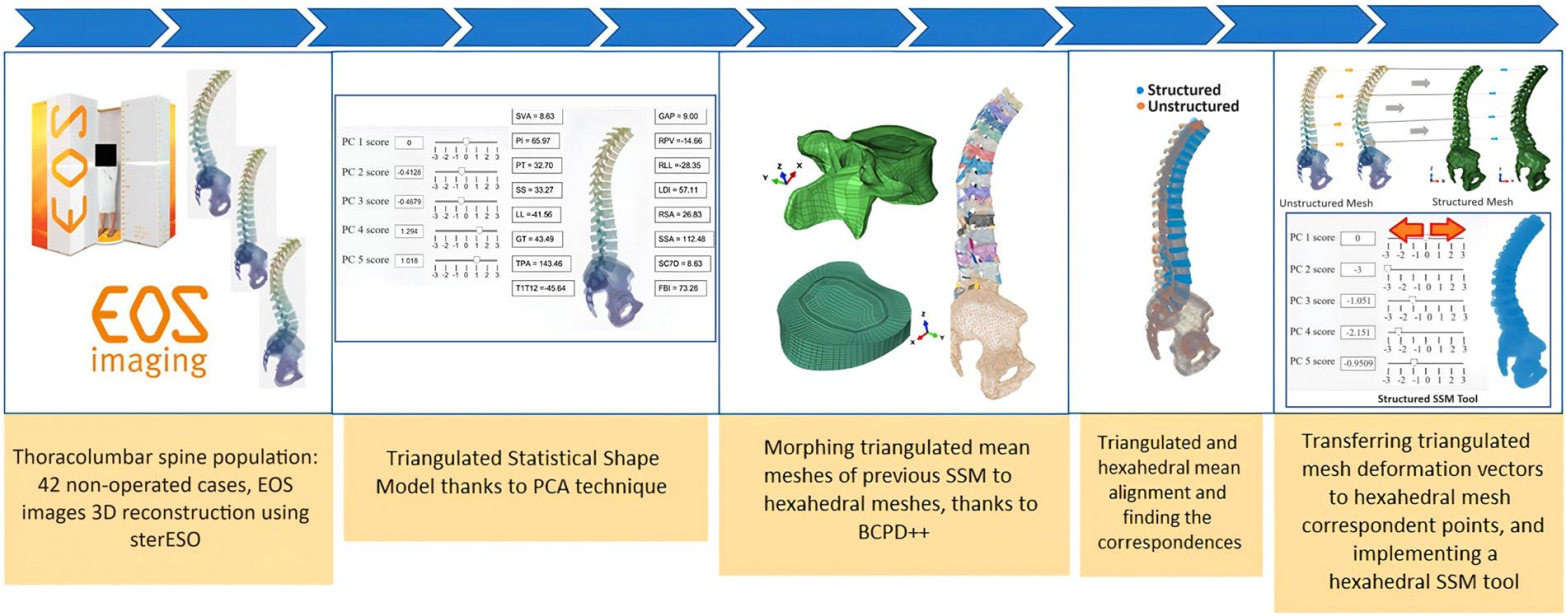
Pipeline to generate FE thoracolumbar spine meshes.

To overcome this limitation, we report an automatable pipeline to build hexahedral thoracolumbar FE spine models out of triangulated surface meshes. Hereby, the triangulated meshes were obtained from EOS 3D information, to allow the modelling of an extensive anatomical region, including the thoracolumbar spine, the sacrum, the pelvis, and the femoral head. We used SSM and the morphing of pre-existing structural meshes developed and verified for the osteo-ligamentous spine^18^, to eventually achieve personalised models that can be used to explore the biomechanics of the thoracolumbar spine, both in balanced and adult spine sagittal deformity. Thanks to the SSM, the full FE modelling pipeline (Fig. 1) allowed to create a virtual cohort of 16807 models through the coupling of different shape modes.

As such, the aim of this article is to share an extensive dataset of ready-to-use structural FE models of the thoracolumbar spine, including (i) a set models personalised against real medical images, and (ii) a set of models that stand for a virtual FE cohort. The model database is further annotated according to state-of-the-art clinical parameters used to quantify the spine geometry and sagittal balance. Overall, the bi-planar EOS images of 42 pre-operated patients affected by adult spine deformity were gathered and converted into 3D surface shape models aligned by rigid registration. Then, Principal Component Analyses (PCA) was used to reduce the dimensionality and explore shape modes representing shape variabilities, as well as computing a mean model. The latter was subsequently transformed to a hexahedral FE model with a 3D hexahedral mesh, using mesh morphing techniques^19,20^. At this stage, the IVD and ligament meshes were included, and the SSM deformation fields that resulted from the PCA were applied on the mean osteo-ligamentous spine hexahedral mesh, to achieve the shared virtual spine deformity thoracolumbar FE models, plus a set of well-balanced spine models.

There are several existing datasets for spine FE models. SpineWeb is a repository which shares lumbar and cervical FE models, developed by the University of California, Berkeley^21^. Vertebral Model Repository (VMR) distributes computational models of individual vertebrae, developed by the University of Auckland, New Zealand. Additionally, another repository shares two patient-specific (49 y.o., and 59 y.o., Female) structural FE meshes of the lumbar spine (L1-L5) and models of Functional Spine Units (FSUs)^22^. These FE models were developed out of segmented CT image data, including IVDs and ligaments. Furthermore, lumbosacral spine models were shared in ArtiSynth^23^. This repository holds hexahedral calibrated and validated models of lumbosacral spine including IVDs, ligaments and sacrum, built with the freely available 3D modelling platform ArtiSynth which supports the combined simulation of multibody and FE models. The existing repositories mentioned earlier suffer from certain limitations. They offer only a limited number of patient-specific models, lack validated thoracolumbar osteo-ligamentous FE models, and do not provide sagittal deformity quantification. As a result, there is a need for a comprehensive dataset that addresses these shortcomings. Such a dataset would offer a substantial collection of thoracolumbar osteo-ligamentous FE models, incorporating sagittal deformity quantification, thereby providing a valuable resource in this field.

To the best of our knowledge, the thoracolumbar spine FE model dataset presented in this article is unique. It comprises 16807 3D thoracolumbar spine FE models, generated through statistical shape variations, representing a virtual cohort. These models have been diligently annotated in terms of spine sagittal balance and are made available through the Zenodo repository. Notably, the model inputs validation, the mono-segmental and multi-segmental Range of Motion (ROM) of the FE models validated against *in-vitro* and *in-vivo* experiments, biomechanical responses of soft tissues validated by *in-vitro* experiments, and the quality of the mesh were thoroughly evaluated and reported.

## Methods

### Thoracolumbar spine triangulated SSM

Here, we used SSM^24^ to analyse and capture the variations within a shape family, as well as generate new shapes.

#### Thoracolumbar spine population

In this study, 42 non-operated EOS bi-planar images are included from IRCCS (Orthopaedic Hospital of Galeazzi, Milan). Inclusion criteria for spine deformities are:

- Age: 50 to 75 years
- (Lumbar Lordosis – Pelvic Incidence) > 10°
- Pelvic Tilt > 20°
- Sagittal Vertical Axis > 5 cm

Aligned spine models also meet the age criteria with aligned spinopelvic parameters. Demographic data of included patients is presented in Table 1. Interestingly, they could cover the spine deformity ranges GAP (Global Alignment and Proportion^25^) from 0 to 13.

**Table 1:** Demographic data of 42 included patients.

Gender	Height (m)	Weight (kg)	Clinical Deformity Classification
50%M, 50%F	1.72 ± 0.22	86 ± 23	***Sagittal***: 7 aligned (GAP 0 to 2), 15 moderate deformities (GAP 3 to 6), 20 severe deformities (GAP 7 to 13); ***Scoliosis***: 16 no scoliosis (cobb angle < 10°), 15 moderate scoliosis (20° >cobb angle> 10°, 11 severe scoliosis (cobb angle >20°)

A total of 42 patient-personalised models of the thoracolumbar spine were reconstructed using the sterEOS software. The sterEOS software utilizes the EOS™ X-ray machine from EOS imaging company in Paris, which has the capability to simultaneously capture bi-planar X-ray images. This innovative technology allows for scanning the entire body in an upright, load-bearing position while minimizing radiation exposure with ultra-low doses^26^. The reconstruction process of the 3D spine surfaces involves two steps, as described below:

1. ***Identification of Control Points:*** A specialist user selects eight control points located at the centre of the vertebral bodies in both sagittal and frontal X-ray images. These control points serve as reference points for estimating and drawing the contours of the vertebrae in the two planes. This initial step results in the reconstruction of a preliminary 3D thoracolumbar spine model using triangulated meshes (Fig. 2, step 1).

**Fig. 2:**
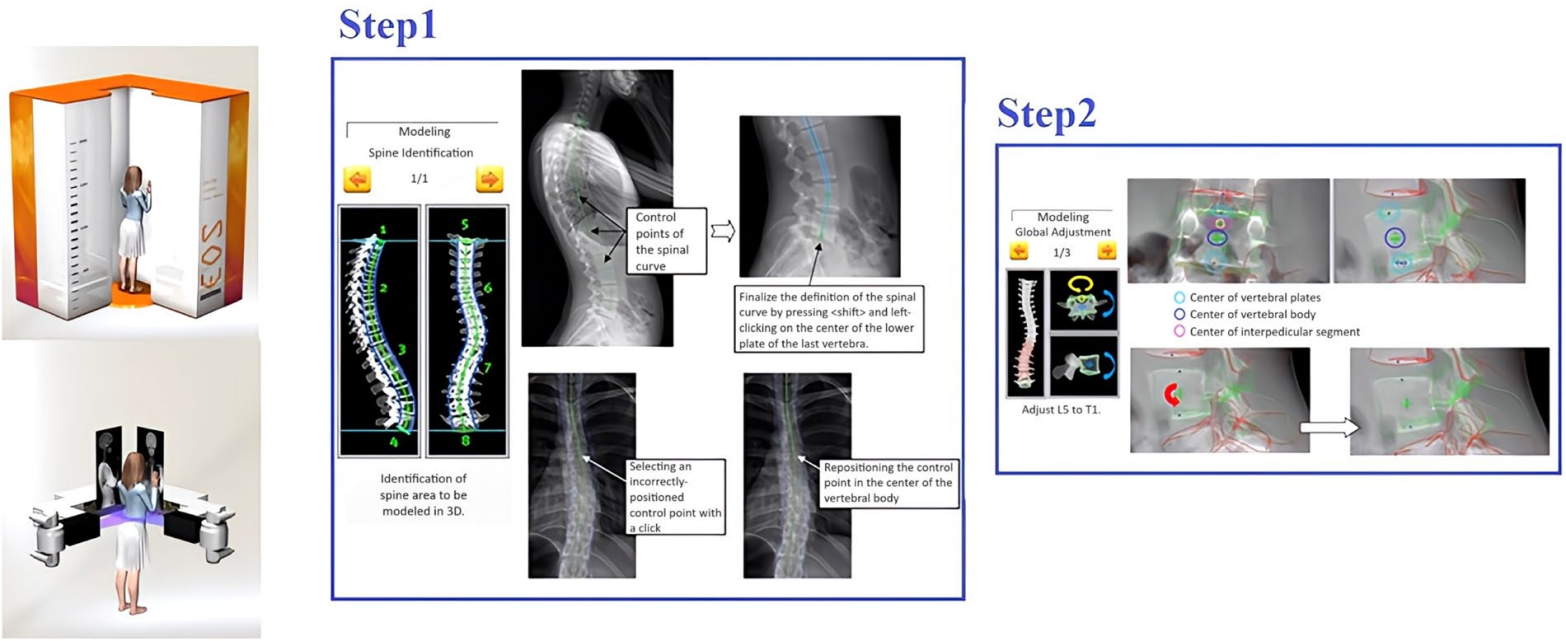
3D reconstruction of thoracolumbar spine models (triangulated meshes) using sterEOS.
2. ***Adjustment of Vertebrae Contours:*** The user can modify the position and orientation of the vertebrae contours to ensure they align accurately with the corresponding bi-planar X-ray images. This step allows for fine-tuning the reconstructed model to achieve a more precise representation of the patient’s spine (Fig. 2, step 2).

42 thoracolumbar spine surface meshes have been saved in the standard DICOM (Digital Imaging and Communications in Medicine) format. To facilitate further analysis and processing, all the 3D DICOM files were converted to the stl format using a C++ code compiled in the Fedora Linux environment. The conversion process was conducted using QT5 Creator and the Mingw environment, with the DICOM to stl converter provided by IRCCS, Milan. Consequently, Stl files representing patient-personalised models of the thoracolumbar spine based on EOS imaging were generated. These stl files are available in a public repository. Additionally, these files were utilized to train the SSM. The spinopelvic parameters were also measured and documented in the accompanying Excel file. To ensure consistency with all patient standing positions, all triangulated spine shapes underwent further alignment.

#### Generalized procrustes alignment (GPA)

All of 42 shapes are aligned rigidly using Generalized Procrustes Alignment (GPA)^27^. Following steps are used for GPA:

- Choosing an arbitrary shape as a reference shape
- Aligning all the shapes to the reference shape
- Computing the mean shape for the aligned shapes
- Considering the mean shape as the new reference shape, and iterating

After aligning the training sets, it is needed to find the correspondences between the points in different models, but all the points in the 42 models are correspondent to each other thanks to EOS imaging system.

#### Developing thoracolumbar spine triangulated SSM

PCA is a widely used technique in shape modelling that is employed in this study to reduce dimensionality and obtain a compact representation of shape variability^28^. PCA serves as a fundamental tool by extracting the principal modes of variation through the computation of eigenvectors derived from the covariance matrix. The covariance matrix, which encapsulates the amount of variation present in the dataset relative to the mean shape, is defined by Eq. 1^28^.

1 $$Cov=\frac{1}{n-1}\mathop{\sum }\limits_{i=1}^{n}\left({X}_{i}-\mu \right){\left({X}_{i}-\mu \right)}^{T}$$

where n is the number of samples, *X*_*i*_ is a matrix including X, Y and Z point coordinates in each sample, and *μ* stands for the average shape which is introduced in Eq. 2.

2 $$Mean=\frac{1}{n}\mathop{\sum }\limits_{i=1}^{n}{X}_{i}$$

Eigenvectors of the covariance matrix are obtained using Singular Value Decomposition (SVD), an operation to decompose a matrix into three matrices, two unitary (eigenvectors) and one diagonal (eigenvalue) matrices. So, the covariance matrix can be decomposed into three matrices in Eq. 3.

3 $$X=\varnothing S{\varnothing }^{T}$$

where ∅ is matrix of eigenvectors, and S is the diagonal matrix of eigenvalues. Then, shapes can be expressed in Eq. 4 as a linear combination of eigenvectors given the feature weights *w*_*i*_^28^.

4 $${\rm{Shape}}={\rm{Mean}}+\mathop{\sum }\limits_{i=1}^{m}\sqrt{{d}_{i}}{\varnothing }_{i}{w}_{i}$$

where m is the number of activated shape modes, *d*_*i*_ is i^th^ diagonal element of matrix S in Eq. 3, ∅_*i*_ is i^th^ column of matrix ∅ in Eq. 3, and *w*_*i*_ is the standard deviation (SD) weight coefficient of activated shape mode, typically *w*_*i*_*∈*{−3, 3}. Hence, each mode in the SSM represents a specific direction of shape variation, which can be visualized as movements of landmarks along straight lines defined by the eigenvectors passing through their mean position. In this study, the eigenvectors and eigenvalues of the covariance matrix are computed using the PCA technique for the set of 42 aligned and corresponding non-operated thoracolumbar spine models. The SSM is then constructed using Eq. 4.

To assess SSM, three techniques have been used: (1) compactness, (2) generalization, and (3) specificity. Here, compactness analysis involved assessing the cumulative variance for different numbers of modes^24^. To determine the optimum number of activated shape modes (m, as defined in Eq. 4), a threshold, such as 95%, is commonly used. The threshold indicates the desired level of explained variance in the training dataset. Results for compactness analysis further showed that the first 5 and 10 shape modes account for 95%, and 98% of the total morphological variation, respectively. Additionally, the individual contribution of each shape mode to the total variation is as follows: mode 1 covers 31%, mode 2 covers 19%, mode 3 covers 18%, mode 4 covers 11%, and mode 5 covers 5%. These findings highlight that shape modes beyond the 5th mode account for less than 5% of the total variation. Therefore, to efficiently represent the morphological diversity in the dataset, the first 5 shape modes were selected for SSM sampling. Moreover, generalization assessed how well the SSM performs on unseen data^24^. Here, the generalization ability was evaluated by performing a series leave-one-out tests on the training sets. To do so, SSM is built using an increasing number of randomly selected training shapes while excluding a target training shape, and then the previously constructed model is then used to reconstruct the excluded shape. The generalization Root Mean Square Error (RMSE) is consequently measured for the average distance between the excluded shape and its reconstructed shape. Here, generalization assessments showed that by increasing the number of training sets from 42 to 50, average generalization RMSE could not be increased significantly (p < 0.05), in the case of including 50 patient training models. It means that SSM was considered population covering when 42 patients were included. Moreover, SSM with 42 training sets and 5 shape modes could have an average generalization RSME of 0.36 ± 0.24 mm which is in the acceptable range (<1 mm) in accordance with the literature^29^. At the end, specificity of SSM is evaluated. The specificity measures the realistic construct of new shape instances randomly generated by the developed shape model^24^. It is measured by generating a large set (N) of virtual shape examples using the constructed model and calculating their difference from real samples available in the training set. The specificity RMSE is also measured here as the distance between the generated shape instance and its most similar sample in the training data. Here, specificity assessments showed that SSM with 42 training sets and 5 shape modes could sample the real spine shapes of the 42 training sets with the average specificity RSME of 0.45 ± 0.21 mm which is in the acceptable range (<1 mm) in accordance with the literature^29^. To sum up, all above evaluations showed that SSM with 42 training sets and 5 shape modes can capture 95% of spine shape variabilities, with the average generalization RSME of 0.36 ± 0.24 mm, and the average specificity RSME of 0.45 ± 0.21 mm. It proves that thoracolumbar spine SSM is a high-performance tool representing different morphologies.

16807 thoracolumbar spine triangulated models (stl files) were sampled using the triangulated SSM tool. The sampling process involved combining the first 5 Principal Components (PCs) and adjusting seven standard deviations (SDs) (-3, -2, -1, 0, 1, 2, 3) for each shape mode. For sampled data, the spinopelvic parameters were measured, and stored in a publicly accessible repository. Moreover, further analysis (see “Supplementary Materials”) showed that these models could cover all the spine deformities.

### Thoracolumbar osteo-ligamentous spine FE hexahedral statistical shape model

Following the pipeline depicted in Fig. 1, the mean shape of the SSM in triangulated meshes is transformed into hexahedral meshes using mesh morphing techniques. To achieve the morphed mean model, the morphing process was applied to both vertebrae and IVDs. The ligaments were incorporated into the model, resulting in the generation of the morphed mean model. Later, the thoracolumbar osteo-ligamentous spine hexahedral SSM was presented.

#### Vertebra mesh morphing

Unfortunately, triangulated meshes coming from images have their unique integration points, which may result in stress discontinuities from element to element, and leading to worse approximations of continuum media^30^. To address that discontinuity issue, hexahedral elements have been replaced. Moreover, triangulated elements can become highly distorted, especially in regions with complex geometries or sharp corners, leading to numerical inaccuracies and convergence issues^31^. In contrast, hexahedral elements tend to have lower distortion, resulting in more stable and reliable simulations. Furthermore, hexahedral elements typically require fewer elements to achieve the same level of accuracy compared to triangulated elements, leading to reduced computational costs and shorter simulation times^32^. Hence, hexahedral meshes are preferred over triangulated elements in spine modelling.

To morph the vertebra meshes, we selected the validated L3 template hexahedral mesh. Subsequently, we morphed the anterior part of the hexahedral vertebra template L3 to match the anterior surfaces of the vertebrae (from L3 to T1) using the accelerated Bayesian Coherent Point Drift (BCPD++) method.

For the thoracic region, it was challenging to morph the posterior elements of the vertebra template L3 to match the posterior thoracic vertebrae due to significant variations in posterior geometry. Therefore, we only morphed the anterior parts of the thoracic vertebrae, while the posterior parts were kept as triangulated elements. Then, a tie constraint between the triangulated posterior and hexahedral anterior elements of thoracic vertebras was defined.

In the lumbar region, given that these shared models are primarily intended for spine orthopaedic surgery and long screw instrumentations, typically involving at least 4 fused levels (from S1 to L3), as elaborated later in the Context of Use (CoU) in the validation steps, we assumed that the entirety of L4 and L5 remains fixed, as lower fused levels are not of interest. Consequently, a tie constraint was defined between the screw and the vertebra, eliminating the necessity for entire hexahedral elements for L4 and L5. Instead, we adapted the validated L3 template quadrilateral mesh to encompass the entirety of L4 and L5. These anterior quadrilateral surface elements for L4 and L5 allow to be matched with the intersegmental IVD hexahedral structures. Furthermore, the inclusion of quadrilateral posterior elements for L4 and L5 enables the precise definition of a facet joint contact model for future studies.

For the posterior elements of L3 to L5, we morphed the validated posterior L3 template quadrilateral meshes to represent the posterior quadrilateral elements of L3 to L1. Then, a tie constraint between the quadrilateral posterior and hexahedral anterior elements of L3 to L1 vertebrae was defined. These quadrilateral posterior elements for L3 to L1 allow to define an accurate facet joint contact model in the future works.

Furthermore, in line with the defined CoUs, it was crucial to incorporate hexahedral elements in the pedicle vertebrae to accurately assess the mechanical behaviour of pedicle screws. Therefore, we utilized Abaqus software (Simulia) to extrude hexahedral meshes from the vertebra bodies to the posterior pedicle for vertebrae L3 to T1.

Here, we preferred to present a complex combination of elements (hexa-, tri-, quad-) to achieve these goals: (1) using previously validated soft tissue structures, and (2) adapting the models to the CoUs. Moreover, in the presence of sufficient computational resources and in consideration of other CoUs, development of full hexahedral thoracolumbar spine models would be valuable in the future works. Moreover, the incorporation of bone property distributions by assigning bone material mapping from CT scan data could provide valuable insights in future studies.

BCPD++ is an extension of the Bayesian Coherent Point Drift (BCPD) algorithm^33,34^. BCPD is a probabilistic method for point set registration^35^, as an extension of the Coherent Point Drift (CPD) algorithm^20^. The CPD algorithm models the transformation between two-point sets using a Gaussian Mixture Model (GMM) and iteratively solves for the optimal transformation parameters^20^. The GMM consists of K Gaussian distributions in the transformation space, and the probability density function of the GMM is defined as^35^:

5 $${\rm{P}}({\rm{Y}}| {\rm{X}},\Theta )=\mathop{\sum }\limits_{j=1}^{K}{{\rm{w}}}_{j}\times {\rm{N}}({\rm{Y}}| {{\rm{\mu }}}_{j},\sum {\rm{j}})$$

where Y is the transformed point set, X is the reference point set, Θ = {w_j_, µ_j_, Σj} is the set of parameters of the GMM, and N(µ_j_,Σj) is the Gaussian distribution with mean µ_j_ and covariance matrix Σj. Then, BCPD computes the correspondence probability between a point x in the reference point set and a point y in the transformed point set as^35^:

6 $${\rm{P}}({\rm{x}},{\rm{y| X}},{\rm{Y}},\Theta )={{\rm{w}}}_{j}\times {\rm{N}}({{\rm{y| \mu }}}_{j},\sum {\rm{j}})$$

where j is the index of the nearest centroid to y; and w_j_, µ_j_, and Σj are the parameters of the corresponding Gaussian distribution. Then, BCPD updates the GMM by re-estimating the centroid locations and the covariances of the Gaussian distributions. It also estimates the posterior distribution of the transformation parameters using Markov Chain Monte Carlo (MCMC) methods. The posterior distribution is given by Bayes’ rule as:

7 $${\rm{P}}\left(\Theta | {\rm{X}},{\rm{Y}}\right)={\rm{P}}\left({\rm{Y}}| {\rm{X}},\Theta \right){\rm{P}}\left(\Theta \right)/{\rm{P}}\left({\rm{Y}}| {\rm{X}}\right)$$

where P(Θ) is the prior distribution of the parameters, and P(Y|X) is the normalization constant. The posterior distribution in BCPD provides valuable information about the uncertainty associated with the estimated transformation parameters. By incorporating a prior distribution, usually a Gaussian distribution, on the transformation parameters, the algorithm becomes more robust against noise and outliers. The estimated posterior distribution is then utilized in BCPD to further refine the transformation parameters, either by computing the mean or the mode of the distribution. The BCPD algorithm iterates until convergence is achieved, or a maximum number of iterations is reached. This iterative process allows for the optimization of the transformation parameters based on the available data and the underlying statistical models.

In this study, the BCPD++ open-source codes are downloaded from the GitHub repository^36^ and used to perform the morphing of the template vertebra. The morphing process utilizes a multi-layer BCPD++ approach, where if the initial morphing yields a Euclidean distance greater than 0.04 mm, the prior output is considered as the template mesh, and the BCPD++ parameters are fine-tuned again. This process is repeated until the Euclidean distance falls below 0.04 mm. To enhance performance and reduce computational time, an initial rotation and rigid registration are applied to the source. The template mesh undergoes a 90° rotation about the y-axis for the initial rotation. Rigid registration is performed using the available BCPD++ code^36^ with a sufficiently large lambda value, such as 1e9. Lambda is one of the tuned parameters in BCPD++, controlling the expected length of deformation vectors. For the BCPD++ non-rigid registration, the initial parameters are specified as shown in Table 2.

**Table 2:** Initial parameters for BCPD++ non-rigid registration.

Parameters	Values
Interaction between the points, Lambda	2
Motion smoothing weight, Lambda	8
Randomness of the point matching at the beginning of the optimization, g	2
Nystrom samples for computing G, K	70
Nystrom samples for computing P, J	300
Scale factor of sigma that defines areas to search for neighbours, d	7
Maximum radius to search for neighbours, e	0.20
The value of sigma at which the KD tree search is turned on, f	0.20
Downsample radius, r	0.50
Expected percentage of outliers, Outlier Ratio	0.10
Maximum number of iterations	1000
Tolerance between consecutive CPD iterations	1e-15

**Fig. 3:**
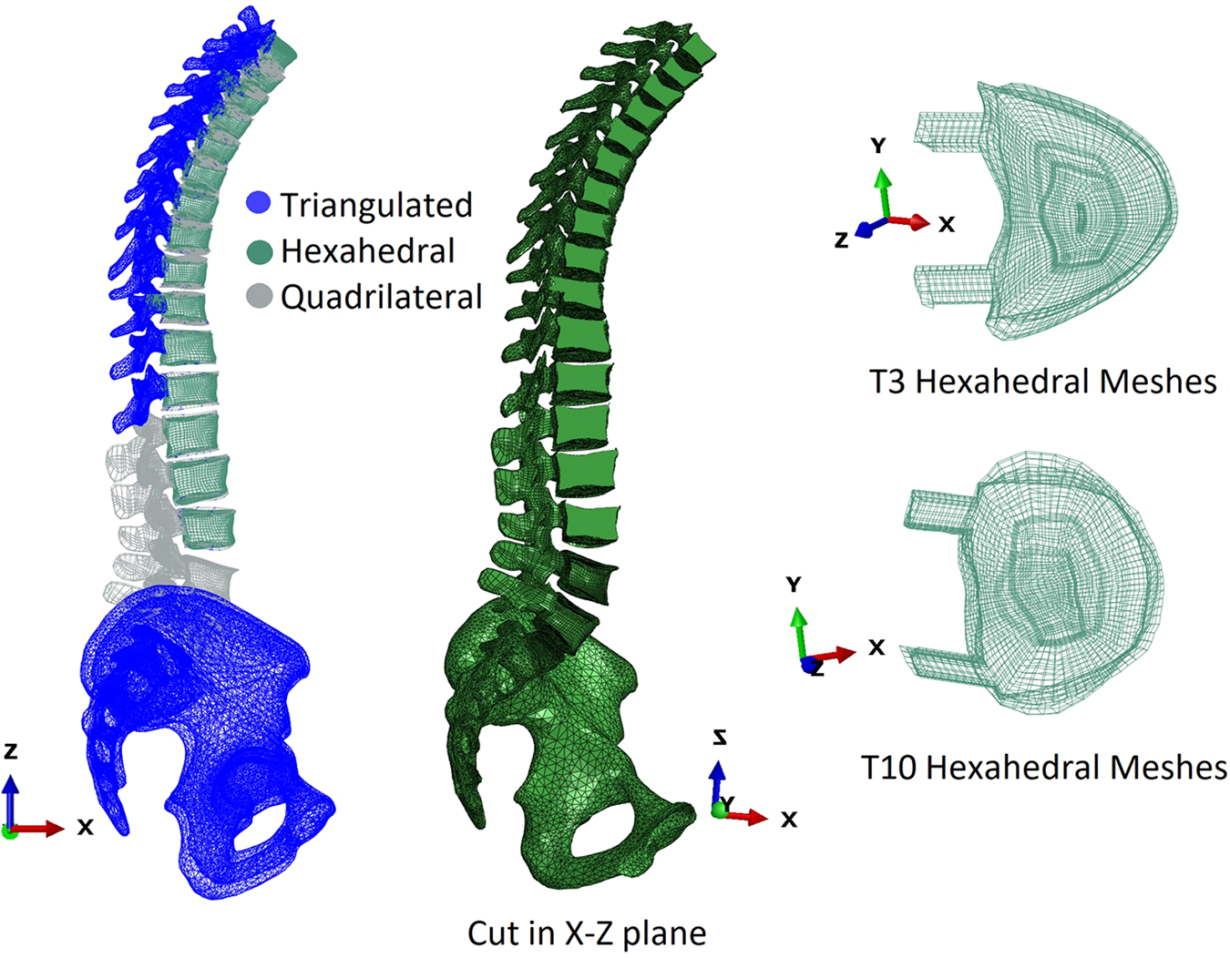
Vertebra mesh morphing process.

Therefore, the morphing process involves converting 15 anterior triangulated vertebras (from L3 to T1) into hexahedral meshes using BCPD++, while 3 posterior triangulated vertebras (L3 to L1) and 2 entire L4 and L5 triangulated vertebras are morphed into quadrilateral meshes using BCPD++. The sacrum or pelvis, as well as the posterior elements of T1 to T12, remain as triangulated meshes. The vertebra mesh morphing process is presented in Fig. 3.

#### IVD mesh morphing

**Fig. 4:**
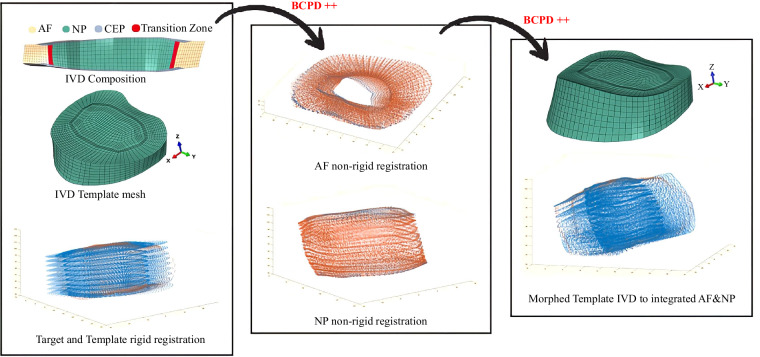
Stepwise IVD L5/L4 morphing process.

Low-dose EOS images lack the precision to accurately measure IVD anatomy, such as the proportions of the Annulus Fibrosus (AF) and Nucleus Pulposus (NP). Hence, IVD surface meshes were not included in the stl files; therefore, quadrilateral meshes are projected onto the two adjacent endplates of vertebras. The outer endplate’s adjacent points are connected to create surrounded quadrilateral elements. These elements are then divided into 8-layer quadrilateral meshes. This process generates the outer IVD quadrilateral meshes in Abaqus software (Simulia). Here, IVD height is patient-specific because up and down surface was adjusted during EOS 3D reconstruction process. Considering this hollow IVD as target, provides this opportunity to morph IVD template (healthy and generic) hexahedral mesh (L4/L3) to the target IVD quadrilateral mesh (Fig. 4).

The IVD template model underwent prior validation through a comprehensive mesh convergence analysis of the generic IVD model which ensured the continuity of strain fields as discussed in the literature^37^. It broadens the CoU for IVD models in the field of biomechanics, thereby avoiding inappropriate discontinuities. Additionally, the ROM of the IVD mesh template in the lumbar spine was validated in the literature^38^. Furthermore, morphing process of the IVD FE mesh template to the target IVD surfaces provided 169 patients specific IVD models which were validated and deposited^39^. This IVD repository facilitates a comprehensive understanding of the mechanisms underlying IVD degeneration. Furthermore, the entire mesh of the IVD template contains 83,481 nodes, with the disc tissues—namely AF, NP, and Cartilage Endplate (CEP)—discretized with 19,392 second-order hexahedral elements (20 nodes). As previously discussed in the literature^37^, this IVD template model represents a more accurate mechanical behaviour of the natural tissues. Additionally, the average thickness of the CEP in the IVD template is 0.7 mm. The NP proportion is set at 40% of the total IVD volume, consistent with literature discussing healthy IVD anatomy^40–44^. Similarly, the NP represents 25% of the transverse cross-sectional area, aligning with the literatures^45–47^.

The BCPD++ morphing technique is further employed to tailor the IVD FE mesh template to fit non-rigidly to the IVD external surfaces, expanded for all the IVD levels in the thoracolumbar spine. The multi-layer BCPD++ mesh refinement technique is utilized for IVD morphing. Similarly, an initial rotation of 90° about the y-axis is applied to the IVD template mesh. BCPD++ rigid registration with a large lambda value (e.g., 1e9) is used to roughly align the IVD target and template. BCPD++ non-rigid registration is then performed using the BCPD++ initial parameters from Table 2. This morphing pipeline guarantees the non-convexity of morphed IVD elements, a critical aspect contributing to the accuracy of the simulations. Moreover, each morphed IVD model retains the same node and element numbers, and element connectivity list across all morphed IVD models. Remarkably, during the morphing process, AF and NP volumetric proportions are controlled to maintain AF at 60% and NP at 40%. Additionally, this process ensures that the CEP thickness remains within an acceptable mean range (0.62 ± 0.29 mm^48^).

Consequently, 17 IVD hexahedral meshes are morphed and integrated with the vertebras. Integrating EOS images with CT or MRI images may provide more accurate IVD and NP anatomy for future modelling studies.

#### Thoracolumbar osteo-ligamentous hexahedral mean model

**Fig. 5:**
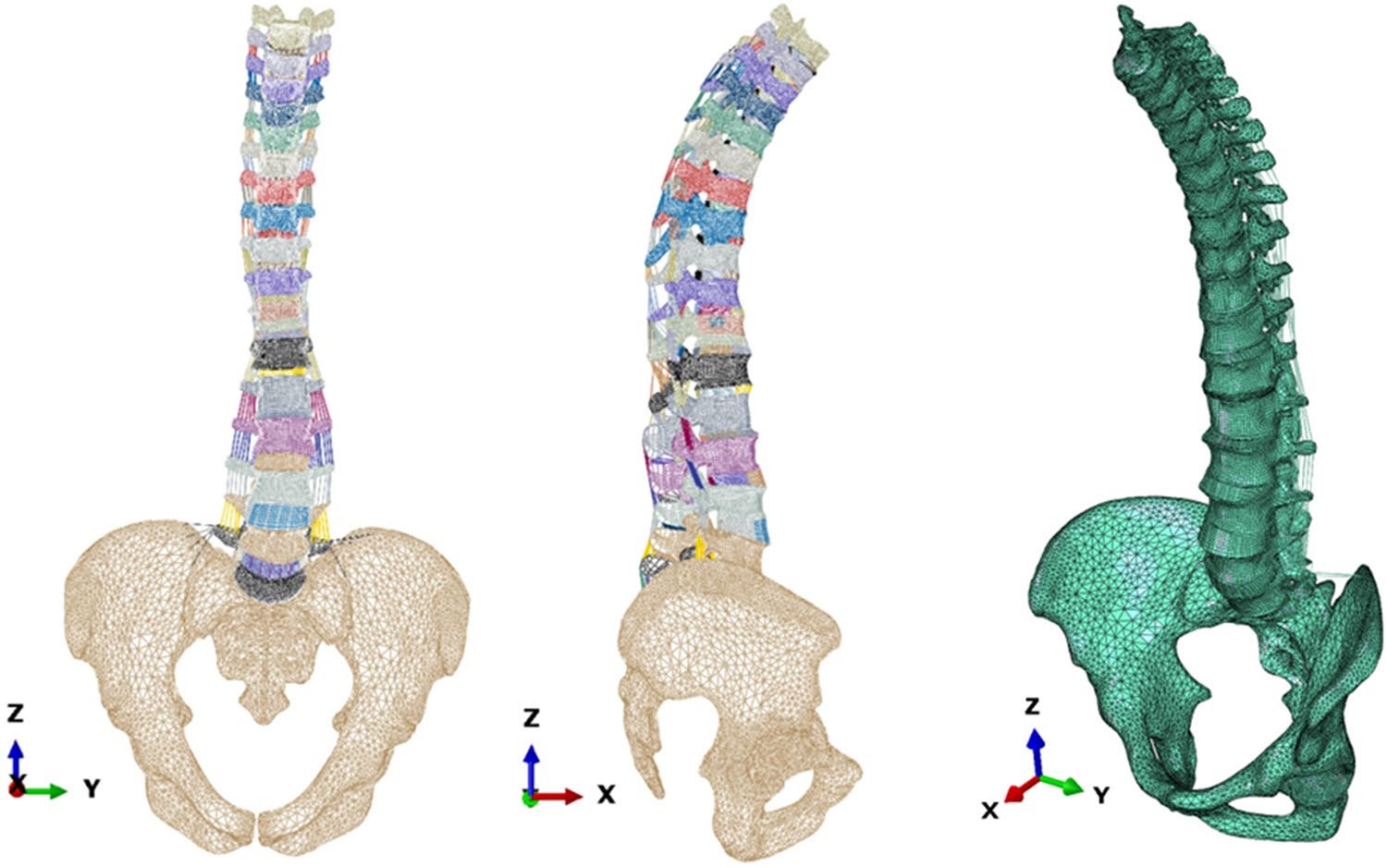
Thoracolumbar osteo-ligamentous hexahedral mean model.

34 thoracolumbar IVDs and vertebras, along with the pelvis, sacrum, and femoral head triangulated meshes, are integrated to create the complete model. Ligaments are modelled as elements by connecting corresponding points within the model. The ligaments are divided into seven groups: Interspinous Ligament (ISL), Supraspinous Ligament (SSL), Ligamentum Flavum (LF), Capsular Ligament (CL), Intertransverse Ligament (ITL), Posterior Longitudinal Ligament (PLL), and Anterior Longitudinal Ligament (ALL). The thoracolumbar osteo-ligamentous hexahedral mean model is depicted in Fig. 5.

**Fig. 6:**
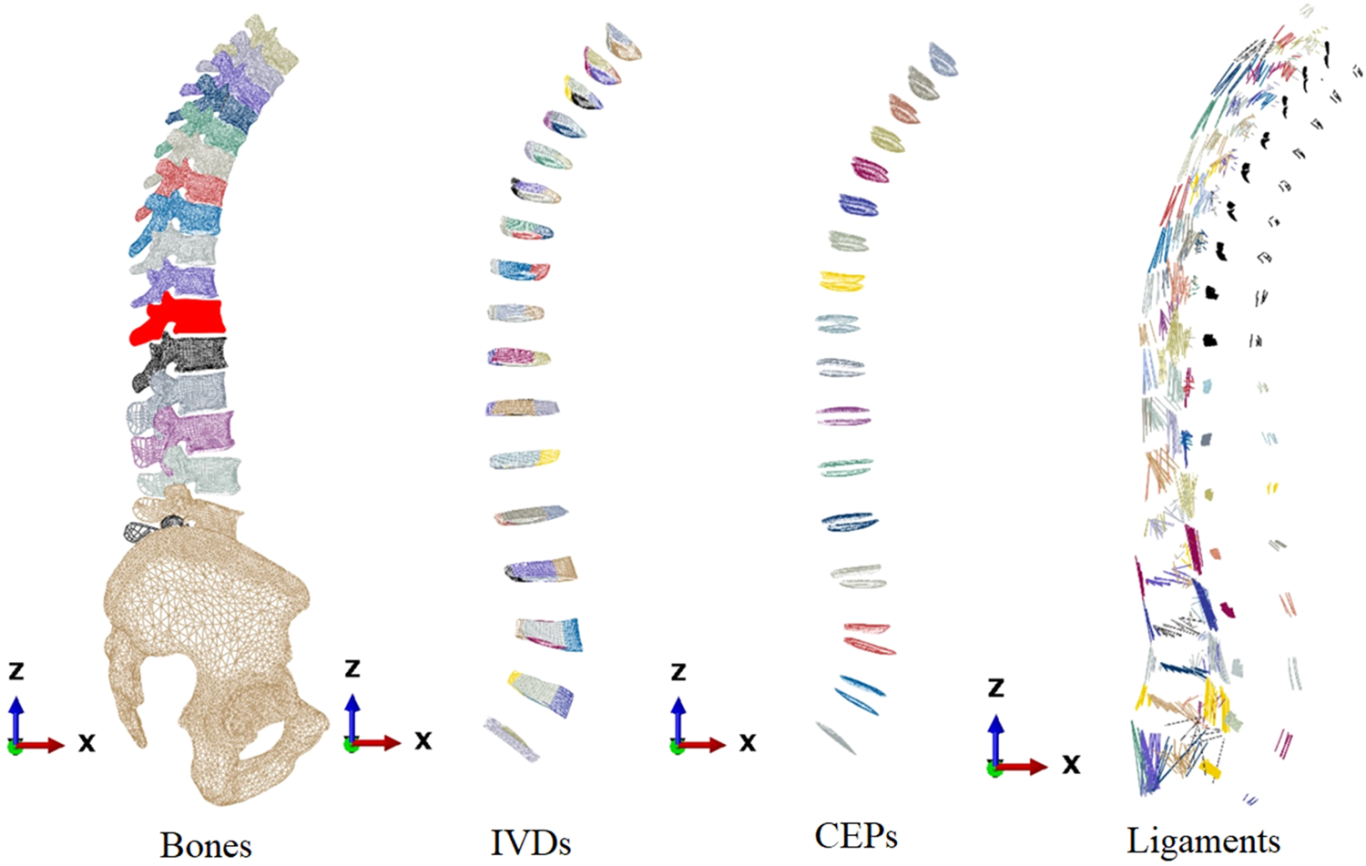
Soft tissue composition in thoracolumbar osteo-ligamentous hexahedral mean model.

Soft tissue composition in thoracolumbar osteo-ligamentous hexahedral mean model is shown in Fig. 6.

#### Developing thoracolumbar osteo-ligamentous spine hexahedral SSM

**Fig. 7:**
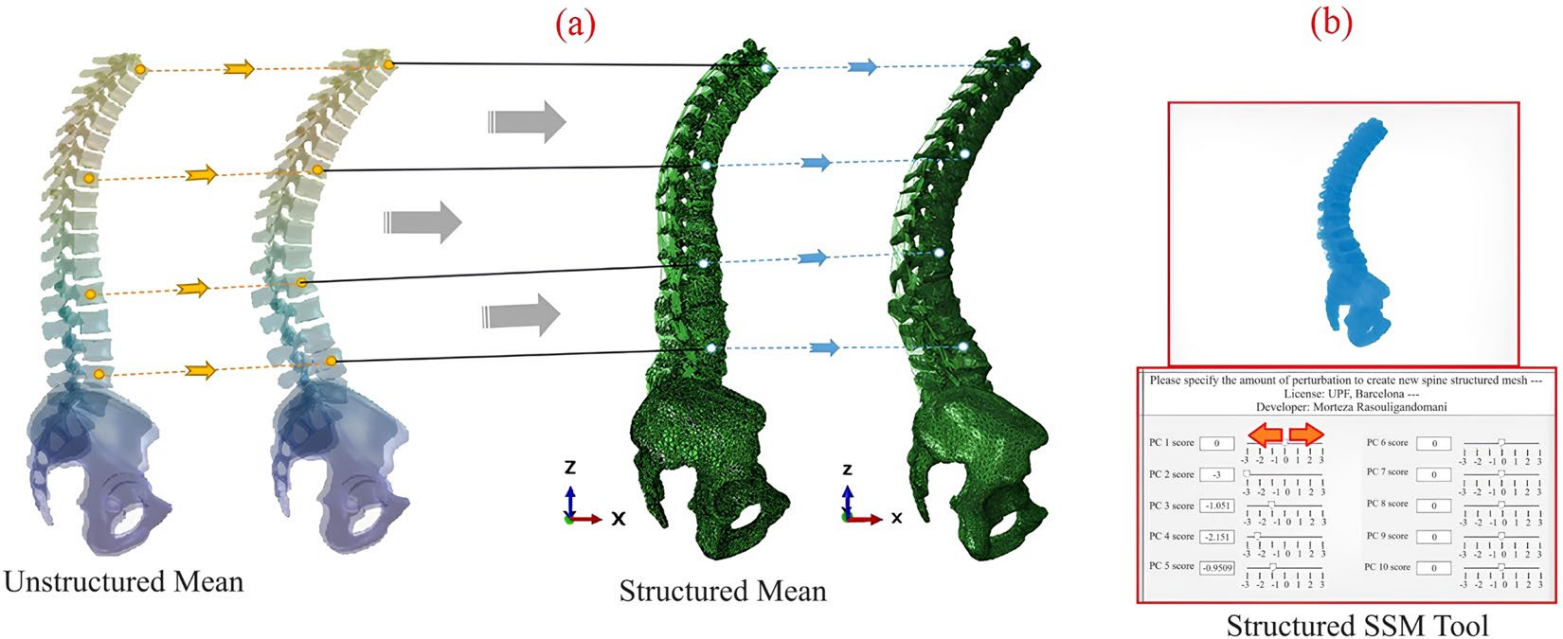
(**a**) Transferring triangulated mesh deformation vectors to hexahedral mesh correspondent points; (**b**) Automatized hexahedral SSM tool.

In the pipeline presented in Fig. 1, the mean hexahedral model is aligned with the mean triangulated model using BCPD++ rigid registration with a large lambda value (1e9). The Iterative Closest Point (ICP) algorithm is then applied to find the closest corresponding points between the two mean models. By iteratively minimizing the distance between the points, the corresponding point IDs are obtained. Equation (4) is updated for the hexahedral SSM by replacing “Mean” with the mean hexahedral model and updating the prior matrix of shape deformations (sqrt(eigenvalues) * eigenvectors) for the corresponding points. This is achieved by considering the deformation vector of a point in the mean hexahedral mesh as the deformation vector of the closest point in the mean triangulated mesh (Fig. 7(a)). Consequently, the updated equation (Shape = Hexahedral_Mean + DF × b) establishes a thoracolumbar spine hexahedral SSM tool (Fig. 7(b)). In this updated equation, DF represents the updated Deformation Fields for the hexahedral meshes, and b corresponds to the SDs ranging from −3 to +3.

**Fig. 8:**
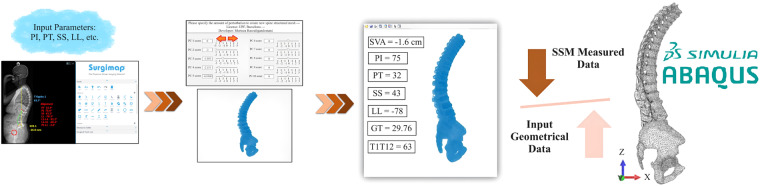
Pipeline to generate FE patient-personalised thoracolumbar osteo-ligamentous spine model using hexahedral SSM tool and input geometrical parameters.

16807 thoracolumbar spine osteo-ligamentous hexahedral FE models, comprising point coordinates, were sampled from the automated hexahedral SSM tool (Fig. 7(b)). This was accomplished by combining the first five PCs and adjusting the shape modes using seven SDs (-3, -2, -1, 0, 1, 2, 3) for each mode. The sampled data, along with the corresponding spinopelvic measurements, is stored in a public repository. Notably, the hexahedral SSM tool (Fig. 7(b)) is independent of image data and relies solely on sagittal parameters of the spine, such as Pelvic Incidence (PI), Pelvic Tilt (PT), Sacral Slope (SS), and Lumbar Lordosis (LL). Geometrical parameters can be measured using clinical software like sterEOS or Surgimap. By activating different shape modes, various spine deformities can be obtained. A comparison between the hexahedral SSM and geometrical data through a pros and cons analysis can facilitate the utilization of patient-personalised FE thoracolumbar osteo-ligamentous spine models (Fig. 8).

**Fig. 9:**
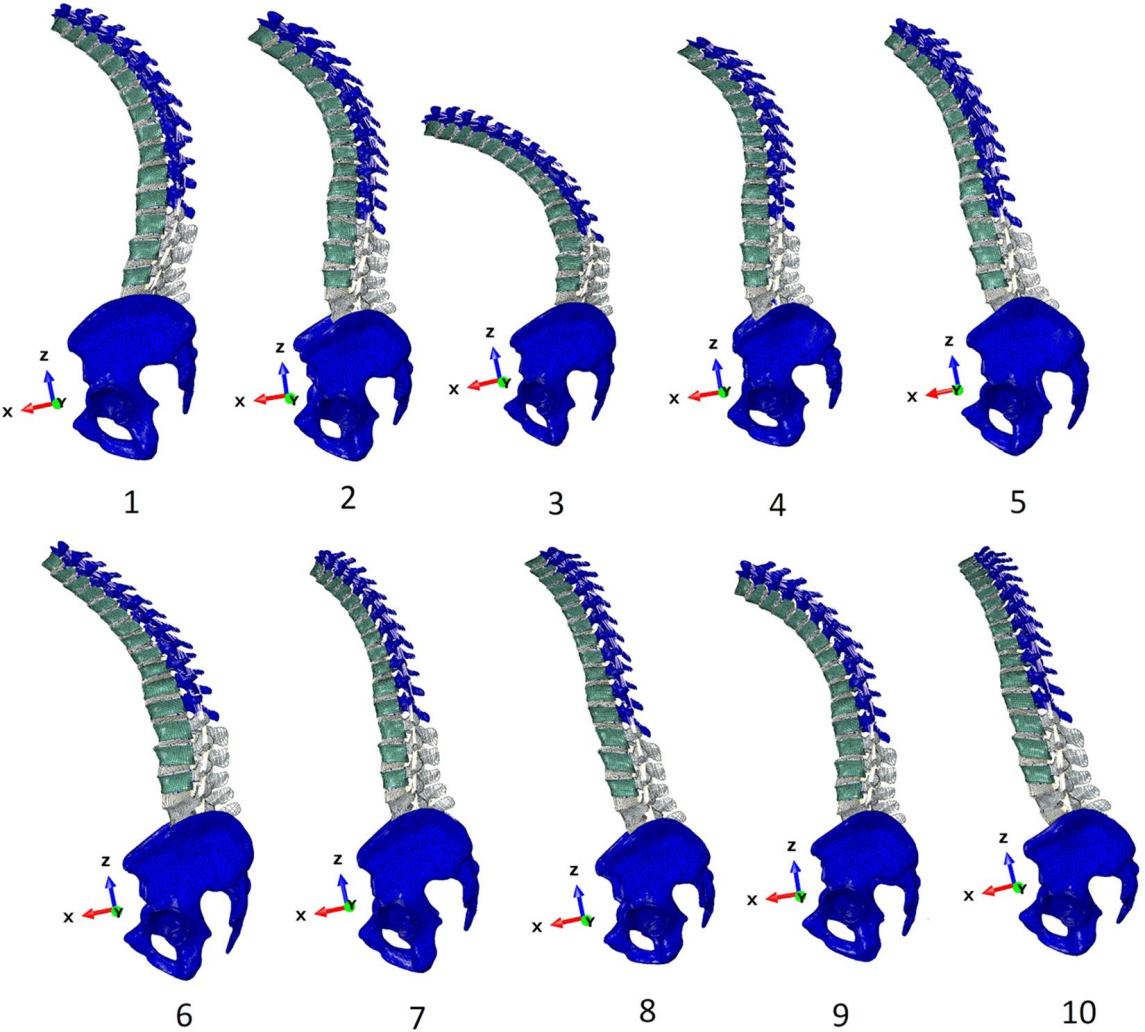
First 10 patient-personalised thoracolumbar spine hexahedral models out of 42 cases.

42 patient-personalised thoracolumbar spine hexahedral models were reconstructed based on the spinopelvic parameters of the included patients (pipeline in Fig. 8). These models have been archived in a publicly accessible repository. Figure 9 showcases the first 10 examples of these patient-personalised thoracolumbar spine hexahedral models.

## Data Records

The pipeline depicted in Fig. 1 involves the sampling of 16807 triangulated and 16807 hexahedral thoracolumbar spine models utilizing the SSM and mesh morphing techniques. Furthermore, we provide 42 triangulated patient-personalised thoracolumbar spine EOS models obtained through sterEOS software, along with their corresponding patient-personalised hexahedral models generated via the SSM. The DICOM images were not shared due to an ethical approval for collected bi-planar EOS images from IRCCS Milan, mentioning that the images could be used for scientific reasons, but sharing the DICOM files of patients was not allowed. All the virtual cohort models and patient-personalised hexahedral models can be generated using 42 shared triangulated patient-personalised thoracolumbar spine EOS models as shared in the current repository. The spinopelvic geometrical parameters were evaluated for each model. All the data, 694GB and consisting of 12.70 billion nodes, are shared in a Zenodo repository as follows:

- ***Thoracolumbar spine triangulated meshes***^49^:
  - 42 stereolithography (stl) files (.stl extension) representing patient-personalised thoracolumbar spine triangulated meshes (“42 Patient-Specific stl files.rar”), including point coordinates and triangulated mesh connectivity IDs. Each stl file includes triangulated meshes of vertebras, pelvis, sacrum, and the femoral head^49^.
  - 16807 stl files representing the thoracolumbar spine triangulated models (“stl.part01.rar” to “stl.part09.rar”), including point coordinates and triangulated mesh connectivity IDs. Each stl file includes triangulated meshes of vertebras, pelvis, sacrum, and the femoral head^49^.
  - An excel file “Descriptive_List.xlsx” reporting measured spinopelvic parameters for 42 patient-personalised triangulated models, and virtual cohort 16807 triangulated models. Spinopelvic parameters are^14^: PI, PT, SS, LL, LL-PI, Global Tilt (GT), Relative Pelvic Version (RPV), Relative Lumbar Lordosis (RLL), Lumbar Distribution Index (LDI), Relative Spinopelvic Alignment (RSA), T1 pelvic Angle (TPA), and scoliosis cobb angle. GAP score^25^ is further measured as a complementary assessment. The Excel file has two sheets: sheet1 for 16807 virtual cohort, and sheet2 for 42 patient-personalised models^49^. Model ID in the excel file is correspondent to the model’s name in both virtual cohort and patient-personalised models^49^. Model number in “Descriptive_List.xlsx” is correspondent to the same model number in 42 and 16807 FE input files, respectively (42 FE input files^50^, 16807 virtual FE input files^51^).
- ***Thoracolumbar osteo-ligamentous spine hexahedral meshes***^50,51^:
  - 42 FE input files (.inp extension, Abaqus software, Simulia) representing patient-personalised thoracolumbar spine hexahedral meshes, including point coordinates, mesh connectivity IDs and element sets. Each input file is almost 99MB (totally 3.86GB), and it includes vertebras and IVDs hexahedral meshes; pelvis, sacrum, and the femoral head triangulated meshes; and ligaments^50^.
  - One png file: “42_P-S.png” representing the first 10 patient-personalised thoracolumbar spine hexahedral models out of 42 FE models^50^.
  - 16807 FE input files representing thoracolumbar spine hexahedral models, including point coordinates. To reduce the size of shared virtual FE models, only point coordinates are shared here. The mean FE input file “Mean_Model (Template).inp” is also shared, which includes point coordinates, mesh connectivity IDs, and element sets. To generate virtual FE input files for any of the 16807 models, the corresponding shared point coordinates can be replaced into the mean FE input file (“Mean_Model (Template).inp”). Mesh connectivity IDs, and element sets are the same in all of FE input files. Mean FE input file includes vertebras and IVDs hexahedral meshes; pelvis, sacrum, and the femoral head triangulated meshes; and ligaments. Each point coordinate file is almost 39MB (totally 655GB)^51^.
  - Two excel files: “Descriptive_List (42_FE_virtual_models^50^; and 16807_FE_virtual_models^51^).xlsx” reporting measured spinopelvic parameters for 42 patient-personalised FE models, and 16807 virtual FE hexahedral models. The Excel file includes measured spinopelvic parameters (PI, PT, SS, LL, LL-PI, GT, RPV, RLL, LDI, RSA, TPA, and scoliosis cobb angle), GAP and IVD centric thickness for FE virtual cohort. Model ID in the excel file is correspondent to the model’s name. Model number in “Descriptive_List (42_FE_virtual_models; and 16807_FE_virtual_models)” is correspondent to the same model number in 42 and 16807 stl files^49^, respectively.
  - One video file: “how_to_replace_point_coordinates.mp4”. It shows how you can replace point coordinates here to the mean FE input file “Mean_Model (Template).inp” in order to generate specific FE input file^51^.

## Technical Validation

The validation of FE models is a crucial step in ensuring their accuracy and reliability. Here, comprehensive validation steps were followed based on the model credibility and the Verification and Validation document, ASME V&V40^52^ (Assessing Credibility of Computational Modelling through Verification and Validation: Application to Medical Devices). Regarding the validation section 5.2 in ASME V&V40, the overall validation compliance was outlined as follows:

1. *Model Form:* While the mathematical formulations of the FE models, including material properties of soft tissues, have been validated in previous work^38^, the model form validation step was omitted.
2. *Model Inputs:* Bi-planar EOS images served as the model inputs for training SSM. Therefore, it was crucial to validate these model inputs.
3. *Test Samples:* This involves comparing the system’s behaviour with *in-vitro* or *in-vivo* experiments. We divided this step into two parts: ROM validation, and FE computational validation. In ROM validation, mono-segmental and multi-segmental ROM of lumbar, thoracic, and thoracolumbar aligned spines (GAP1) were validated against previous *in-vitro* or *in-vivo* experiments. In FE computational validation, aligned spine biomechanical behaviours were validated against previous *in-vitro* results.
4. *Test Conditions:* This refers to testing different conditions that may affect the system outputs. Factors such as different material properties, boundary conditions, and contact models were beyond the scope of this manuscript and may need to be studied in future works.
5. *Equivalency of Input Parameters:* The type and ranges of all inclusion criteria were consistent for all 42 non-operated EOS bi-planar images. Additionally, demographic data (Table 1) for the 42 included data confirmed that the input patients were equivalent (e.g., 50% female, 50% male).
6. *Output Comparison:* Shared datasets in the Zenodo repository were sampled from the SSM tool. However, the traditional method of obtaining these models was through the generation of models from CT images. Ground-truth validation involves comparing models generated by SSM with models segmented and generated from CT images. This step is beyond the scope of this manuscript and may need to be addressed in future works.

In summary, three validation steps compliant with ASME V&V40 have been studied: (1) Model Input Validation, (2) ROM Validation, and (3) FE Computational Validation. These validation steps were reflected in the following sections. Furthermore, defining the CoU for the shared models was essential, with the primary CoU being spine orthopaedic surgery. Sub-CoUs could also be defined as: (1) Virtual surgical planning and simulation; (2) Implant design and evaluation; (3) Spine surgery prediction and prognosis; (4) Spine surgery training and education; and (5) Spine patient-personalised modelling.

### Model input validation

The image datasets and thoracolumbar surface meshes used to train the triangulated SSM were obtained from IRCCS Galeazzi in Milan, utilizing the EOS modality and sterEOS software. Patient selection was based on predefined inclusion criteria. The Spine 3D surfaces were reconstructed semi-automatically using the sterEOS software, with the assistance of an independent expert. The accuracy of the EOS system was thoroughly studied and reported in the original article^26^, which describes the EOS machine and 3D reconstruction using the ster-EOS software. It reports *in-vitro* validation for 36 vertebrae, 25 proximal femurs, 25 distal femurs and 7 tibias. The average error was 0.90 mm, and 95% of the errors were less than 2.40 mm. Moreover, with regards to *in-vivo* validation, and even in the case of very deformed bones (severe scoliosis and arthritic knees), the average error remained less than 1.50 mm. It shows that the EOS accuracy in the sagittal plane is 0.90 mm on average and by changing the plane to the frontal plane, the EOS accuracy was still less than 1.50 mm. All this evidence demonstrates that the accuracy of EOS thoracolumbar spine 3D reconstruction is high and does not significantly impact SSM accuracy. Moreover, ster-EOS software enabled automatic validation of spine morphological parameters, and in case of any out-of-range values, the user had the ability to make necessary amendments to the reconstruction process. Additionally, morphological measurements were carefully reviewed and validated by a senior spine orthopaedic surgeon at Hospital del Mar in Barcelona. This meticulous validation process ensured that the image-based reconstructed models achieved the highest level of accuracy and quality, as they underwent continuous measurements validation. Notably, the geometry re-calibration process was not required in this study, thanks to the utilization of EOS imaging technology.

### ROM validation

**Fig. 10:**
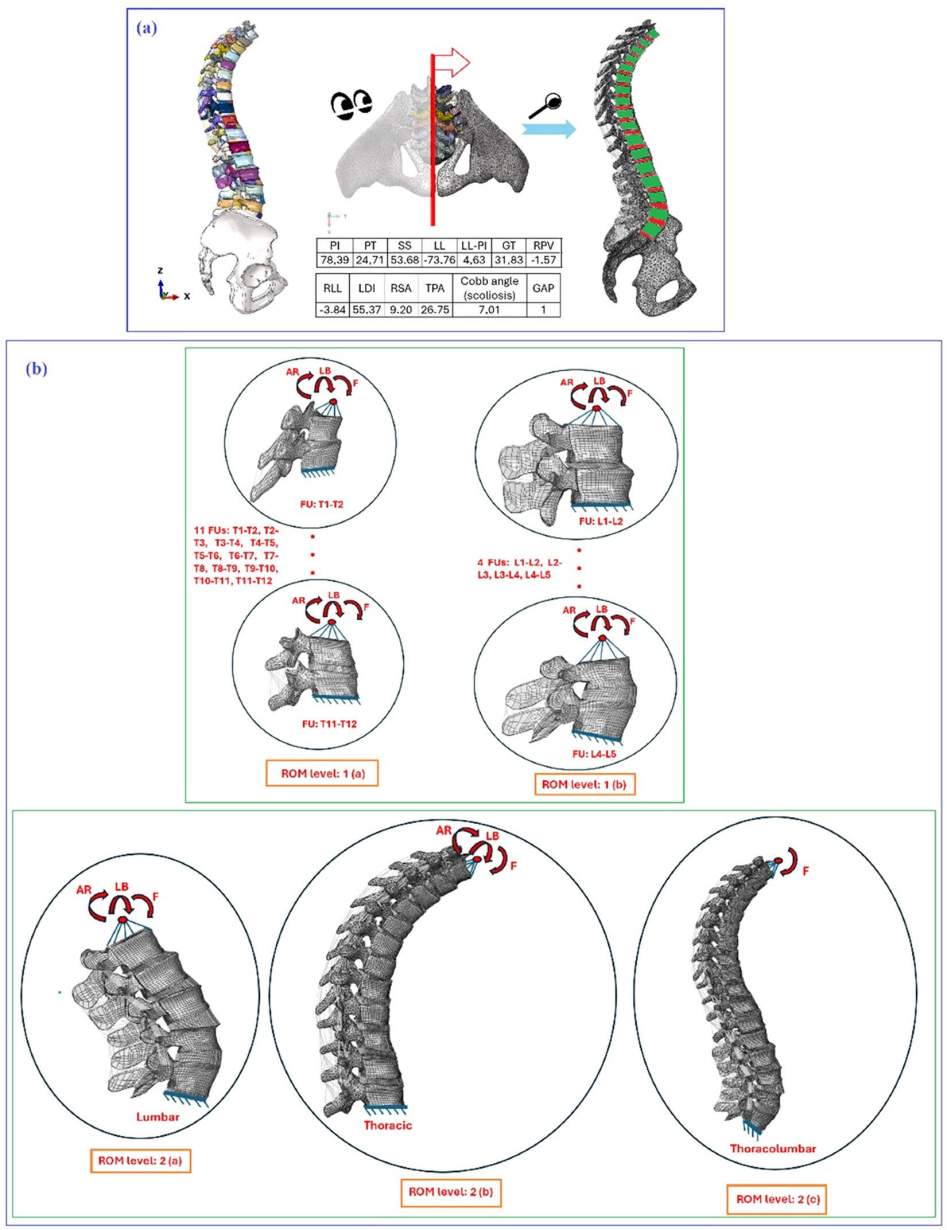
(**a**) An aligned spine model (ID number 17, GAP 1) extracted from 42 shared models^50^ including the geometrical parameters; (**b**) Two levels for ROM: 1- Mono-segmental functional units; 2- multi-segmental for lumbar, thoracic, and thoracolumbar spine (1(a): thoracic functional units, 1(b): lumbar functional units, 2(a): lumbar, 2(b): thoracic, 2(c): thoracolumbar).

ROM analysis is a widely used technique for validating FE models, as it involves measuring the movement and displacement of a physical object. In this study, the mono-segmental and multi-segmental ROM of one aligned spine (GAP 1) FE model was evaluated and compared to previous works for validation purposes. This aligned model (GAP 1), identified by ID number 17, was extracted among 42 patient-personalised thoracolumbar spines hexahedral meshes^50^ (see Fig. 10(a)). Furthermore, Fig. 10(a) presents the spinopelvic parameters (PI: 78.39°, PT: 24.71°, SS: 53.68°, LL: −73.76°, LL-PI: 4.63°, GT: 31.83°, RPV: −1.57°, RLL: −3.84°, LDI: 55.37°, RSA: 9.20°, TPA: 26.75°, cobb angle (scoliosis): 7.01°, and GAP: 1) of this aligned model. The detailed description of constitutive laws was beyond the scope of this paper. However, the material properties and boundary conditions employed for the ROM analyses are briefly outlined in Table 3.

**Table 3:** Material properties and boundary conditions for ROM analyses.

***Material properties***	***IVD***	***Vertebras***	***Ligaments***
	Anisotropic hyperelastic model^40^	Linear elastic^73^	Hypoelastic model^74^
***Boundary Conditions***	Sacrum is fixed, three moments are applied: flexion, lateral bending, and axial rotation		

The ROM of different spine segments and integrated compartments were simulated using Abaqus software (Simulia) on a cluster supercomputer located at Pompeu Fabra University in Barcelona.

Two levels for ROM including their sub-models are introduced:

1. Mono-segmental functional units (Fig. 10(b)):

- All thoracic functional units are selected, then the pure moments are applied to induce Flexion (F), Lateral Bending (LB), and Axial Rotation (AR) in the upper endplates of each functional units. Intersegmental rotation angles (F, LB, and AR) are measured. Results are validated against the average values in an *in-vitro* test^53^. In this *in-vitro* test^53^, a total of 68 thoracic FSUs from 29 human donors (aligned human spines) were tested. The average age of the donors was 57 years (40–80 years), whereby 13 of the donors were male and 16 were female^53^.
- All lumbar functional units are selected, then the pure moments are applied to induce F, LB, and AR in the upper endplates of each functional units. Intersegmental rotation angles (F, LB, and AR) are measured. Results are validated against the average ROM values of three *in-vivo* tests^54–56^. In an *in-vivo* test^54^, lumbar functional units from 11 male volunteers were selected. None of the subjects experienced any back pain or sagittal deformity during the 12-month follow-up. In another *in-vivo* test^55^, lumbar functional units from two groups of 10 male volunteers were included, with one group undergoing axial rotation and the other experiencing lateral bending. Again, none of the subjects reported any back pain or sagittal deformity during the 12-month follow-up. In yet another *in-vivo* test^56^, lumbar functional units from 11, 10, and 10 aligned subjects were selected to test flexion and extension, axial rotation, and lateral bending, respectively.

2. Multi-segmental lumbar, thoracic, and thoracolumbar spine (Fig. 10(b)):

- Lumbar spine (L5-L1) is selected, then the pure moments are applied to induce F, LB, and AR in the upper endplates of L1. Intersegmental rotation angles (F, LB, and AR) for each lumbar functional unit are measured. Results are validated against the average ROM values of female and male patients in an *in-vitro* test^57^. In this *in-vitro* test^57^, 42 human lumbar segments (L1-S1) were subjected to the pure moments of F, LB, and AR, with the mean age of 57.52 years (range 24 to 74 years), mean height of 1.69 m, and mean weight of 97.66 kg^57^.
- Thoracic spine (T12-T1) is selected, then the pure moments are applied to induce F, LB, and AR in the upper endplates of T1. Thoracic flexion (presented in Table 4), axial rotation, and lateral bending angles are measured. Results are validated against the average ROM values of whole T12-T1 segment without rib cage in an *in-vitro* test^58^. In this *in-vitro* test^58^, six human thoracic spine specimens, including the entire rib cage, were loaded with pure moments of F, LB, and AR using a custom-built spine tester. The average age was 56 years (range 50 to 65 years), with one specimen from a male volunteer and five specimens from female volunteers^58^. No misaligned thoracic spine specimens were observed^58^.

**Table 4:** ROM results for different FE models (1(a): thoracic functional units, 1(b): lumbar functional units, 2(a): lumbar, 2(b): thoracic, 2(c): thoracolumbar), and validation against *in-vitro* or *in-vivo* experiments; *Acronyms: N.m: Newton meter, TK: Thoracic Kyphosis*.

ROM level: 1(a)			ROM Level: 1(b)			ROM Level: 2(a)			ROM Level: 2(b)		ROM Level: 2(c)	
FU	FE results for bending 7.50 N.m	Average *In-vitro* results^53^	FU	FE results for bending 7.50 N.m	Average *In-vivo* results^54–56^	FU	Lumbar FE results for bending 7.50 N.m	Average *In-vitro* results^57^	Thoracic FE results for bending 2 N.m	Average *In-vitro* results^58^	Thoracolumbar FE results for bending 2 N.m	Average thoracolumbar flexion angle^59^
T1-T2	F: 7.50°, LB: 5.60°, AR: 5.80°	F: 7°, LB: 5.90°, AR: 6.10°	L1-L2	F: 7°, LB: 5°, AR: 1°	F: 6°, LB: 4°, AR: 1°	L1-L2	F: 5°, LB: 7°, AR: 3°	F: 7°, LB: 7°, AR: 2.50°	F: 18°, LB: 25°, AR: 44°	F: 16°, LB: 23°, AR: 46°	F: 16.20°	F: 17.40°
T2-T3	F: 3.80°, LB: 4.70°, AR: 5°	F: 4.10°, LB: 4.90°, AR: 5°	L2-L3	F: 11°, LB: 5°, AR: 1.50°	F: 10°, LB: 5°, AR: 1.10°	L2-L3	F: 8°, LB: 13°, AR: 4°	F: 8°, LB: 9°, AR: 4°				
T3-T4	F: 3.60°, LB: 5.60°, AR: 5.60°	F: 3.80°, LB: 5.80°, AR: 5.90°	L3-L4	F: 12.50°, LB: 6°, AR: 1.40°	F: 12°, LB: 5°, AR: 1.20°	L3-L4	F: 8°, LB: 12°, AR: 4°	F: 9°, LB: 10°, AR: 5°				
T4-T5	F: 3.20°, LB: 4.70°, AR: 4.80°	F: 3.30°, LB: 4.90°, AR: 5.10°	L4-L5	F: 15°, LB: 4°, AR: 1.40°	F: 15°, LB: 3°, AR: 1.20°	L4-L5	F: 13°, LB: 7°, AR: 4°	F: 11°, LB: 9°, AR: 4°				
T5-T6	F: 3.70°, LB: 5.10°, AR: 5.60°	F: 3.90°, LB: 5.10°, AR: 5.80°										
T6-T7	F: 3.80°, LB: 5.10°, AR: 5.80°	F: 3.93°, LB: 5.20°, AR: 6°										
T7-T8	F: 2.60°, LB: 3.70°, AR: 4.50°	F: 2.80°, LB: 3.80°, AR: 4.70°										
T8-T9	F: 3.10°, LB: 3.90°, AR: 4.70°	F: 3.20°, LB: 3.90°, AR: 4.90°										
T9-T10	F: 3.50°, LB: 4.30°, AR: 4.90°	F: 3.60°, LB: 4.50°, AR: 5.10°										
T10-T11	F: 2.87°, LB: 3.70°, AR: 4°	F: 3°, LB: 3.90°, AR: 4°										
T11-T12	F: 3.50°, LB: 3.70°, AR: 3.10°	F: 3.60°, LB: 3.80°, AR: 3.30°
- Thoracolumbar spine (L5-T1) is selected, then the pure moment is applied to induce F, LB, and AR in the upper endplates of T1. Thoracolumbar flexion angle^59^ (presented in Table 4) is measured. Results are validated against the average thoracolumbar flexion angle reported in a literature^59^. In this trial test^59^, the following specimens were included: 15 subjects with aligned sagittal spines (9 females and 6 males), with an average age of 23.6 ± 2.0 years. The mean height and weight were 1.57 ± 0.04 m and 61.5 ± 5.2 kg, respectively. The subjects performed lumbar flexion-extension tasks, after which the thoracolumbar flexion angle was measured^59^. This angle was defined as the angle between a line from the T9 spinous process to the T12 spinous process and a line from the T12 spinous process to the L2 spinous process^59^. The thoracolumbar flexion angle was measured and recorded using a digital camera and the video motion analysis software Pro-trainer (ver. 10.1; Sports Motion, Cardiff, CA, USA)^59^.

ROM results and validations against the *in-vitro* or *in-vivo* experiments are summarized in Table 4.

Results in Table 4 showed that F, LB, and AR of thoracic functional units in an aligned spine had the maximum percentage difference of 7.41%, 5.26%, and 6.25%, respectively, compared to the average *in-vitro* results. Moreover, F, LB, and AR of lumbar functional units in an aligned spine had the maximum percentage difference of 15.38%, 28.57%, and 30.76%, respectively, compared to the average *in-vivo* results. Intersegmental F, LB, and AR of lumbar L5-L1 model had the maximum percentage difference of 15.38%, 28.57%, and 30.76%, respectively. F, LB, and AR of thoracic T12-T1 had the percentage difference of 11.76%, 8.33%, and 4.44%, respectively, compared to the average *in-vitro* results. Also, flexion of thoracolumbar spine had the percentage difference of 7.14% compared to the average thoracolumbar flexion angle. All these results confirm that the ROM of an aligned FE model (model’s ID 17 in 42 shared models^50^, GAP 1) is close to the experiments.

**Fig. 11:**
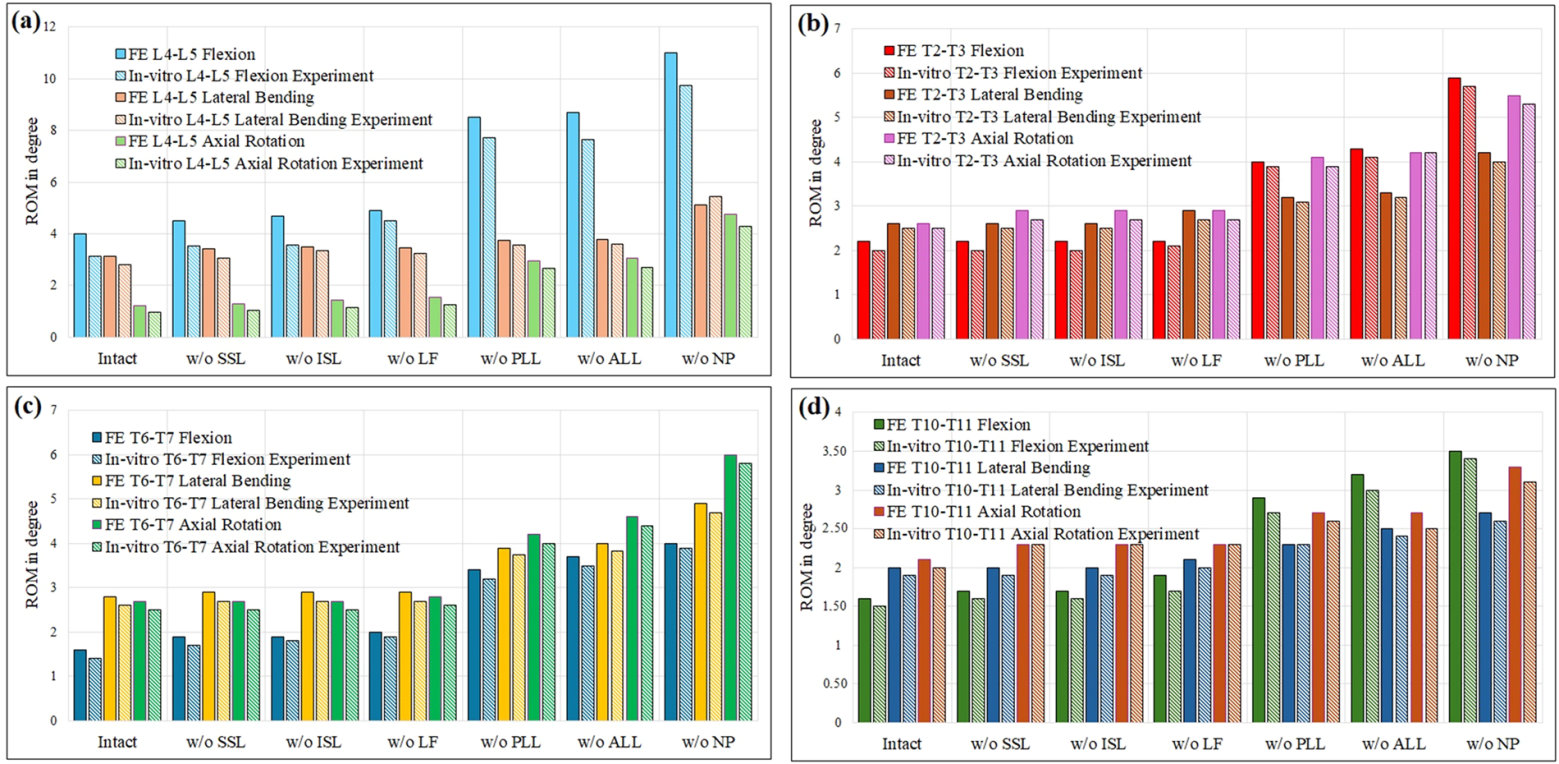
(**a**) FE L4–L5 ROM of stepwise reduction of functional spinal structures (aligned model with ID number 17 extracted from 42 shared models^50^, GAP 1), compared with *in-vitro* experiment in the literature^60^; (**b**) FE T2–T3 ROM of stepwise reduction of functional spinal structures, compared with *in-vitro* experiment^61^; (**c**) FE T6–T7 ROM of stepwise reduction of functional spinal structures, compared with *in-vitro* experiment^61^; (**d**) FE T10–T11 ROM of stepwise reduction of functional spinal structures, compared with *in-vitro* experiment^61^; *Acronym: w/o: Without*.

Additionally, the ROM resulting from the stepwise reduction of functional spinal structures was measured in the L4–L5 lumbar model (aligned model with ID number 17, extracted from 42 shared models^50^, GAP 1), as well as in the T2-T3, T6-T7, and T10-T11 thoracic models under a bending load of 2.50 N.m, as depicted in Fig. 11. These results are also compared with those from *in-vitro* tests^60,61^. In the *in-vitro* test^60^, eight lumbar spinal segments (L4–L5), with a mean age of 52 years (ranging from 38 to 59 years) and no signs of disc degeneration, were utilized. The specimens were subjected to pure moments (ranging from 1 to 10 N.m) in the three principal anatomical planes. Subsequently, the anatomy was stepwise reduced by cutting various ligaments and removing the nucleus. In another *in-vitro* test^61^, six fresh-frozen human thoracic spinal motion segments at levels T2–T3, T6–T7, and T10–T11, respectively, were harvested from nine human aligned spines. The donors had an average age of 58 years, ranging from 50 to 65 years^61^. These specimens were loaded with pure moments of F, LB, and AR^61^. After each loading step, ligaments and the nucleus were stepwise resected in a posterior-to-anterior direction, while segmental relative motions were measured using an optical motion tracking system^61^.

Overall, results in Fig. 11 confirm that FE ROM of stepwise reduction of functional spinal structures for lumbar and thoracic segments of an aligned model (model’s ID 17 in 42 shared models^50^, GAP 1) is close to *in-vitro* experimental results, which highlights the ROM validity of the shared models.

### FE Computational validation

An aligned FE osteo-ligamentous thoracolumbar spine model (model’s ID 17 in 42 shared models^50^, GAP 1) was selected. Then, the mechanical responses of ligaments and IVDs were validated in two FE computational tests, compared with literature, *in-vivo*^62^, and *in-vitro* tests^63^.

#### Ligaments mechanical response

**Fig. 12:**
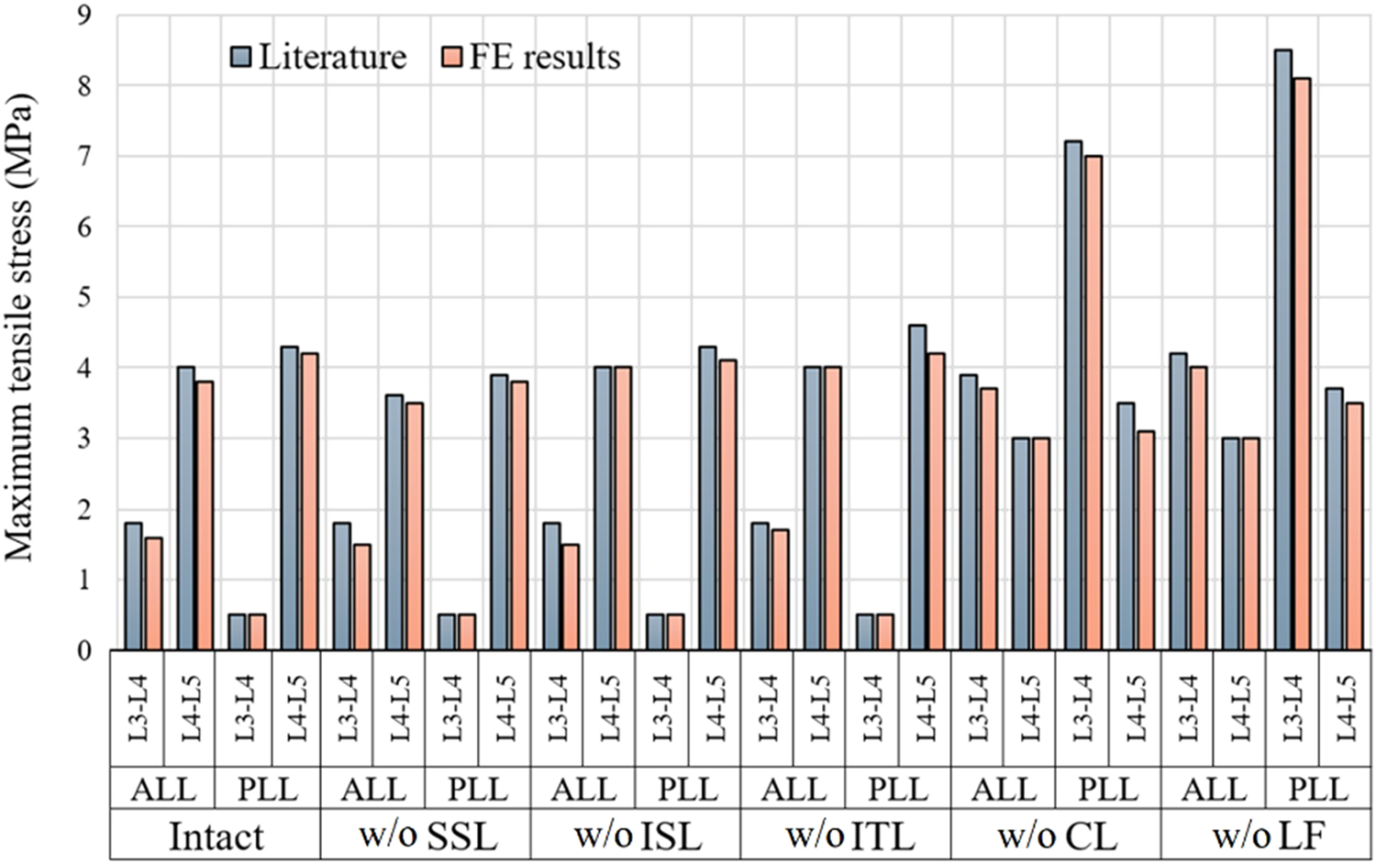
Ligament tensile stresses for the intact version of aligned (model’s ID 17 in 42 shared models^50^, GAP 1) L3-L5 model and after each successive resection in flexion at 7.5 N.m, results compared with literature^38^; *Acronym: w/o: Without*.

Here, a L3-L5 lumbar spine model was isolated. Then, ligament tensile stresses were evaluated after each successive ligament resection in flexion at 7.50 N.m. Results were further compared with literature^38^ and presented in Fig. 12.

Results in Fig. 12 showed that maximum percentage difference between FE and literature results for ligament stress was 18.18% which showed that ligaments mechanical response was close to the literature^38^.

#### IVD mechanical response

Here, two mechanical tests in lumbar and thoracic part were performed.

In the lumbar part, an IVD L4-L5 was selected (aligned model with ID number 17 in 42 shared models^50^, GAP 1), and a FE computational test was designed based-on an *in-vivo* experiment^62^. IVD L4-L5 was loaded corresponding the weight of 70 kg for a patient. Swelling step has been defined in IVD for 24 hours and in a relax standing. Then, IVD Intradiscal Pressure (IDP) has been computed in FE simulation. In contrary, in the *in-vivo* test, under sterile surgical conditions, a pressure transducer with a diameter of 1.50 mm was implanted in the NP of a non-degenerated L4-L5 IVD of a male volunteer 45 years and weighing 70 kg^62^. IVD pressure was recorded with a telemetry system during a period of approximately 24 hours for relaxed standing^62^. FE computational results showed the average IDP of 0.47 MPa which was in accordance with *in-vivo* results with IVD pressure of 0.50 MPa in literature^62^ (6.18% difference). These results proved that the IVD L4-L5 mechanical response was in accordance with the *in-vivo* test and by expanding validated IVDs to all lumbar levels, it reflects the validity of lumbar ROM behaviour.

**Fig. 13:**
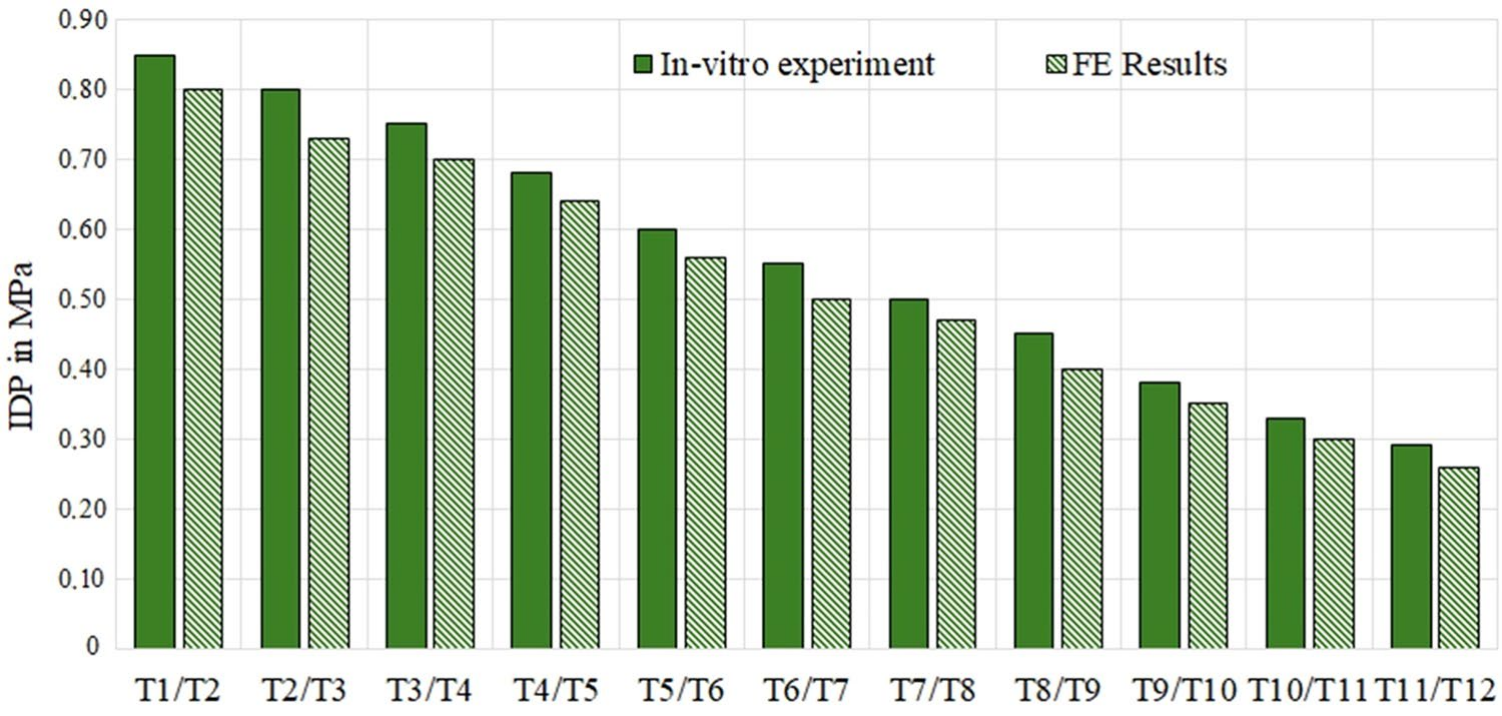
IVD IDPs for thoracic functional units underwent flexion of 7.50 N.m in an aligned spine model (model’s ID 17 in 42 shared models^50^, GAP 1), results compared with literature^63^; *Acronyms: IDP: Intradiscal Pressure*.

In addition, IDPs in the thoracic spine (aligned model with ID number 17 in 42 shared models^50^, GAP 1) were measured by FE simulations and compared with *in-vitro* results^63^. Here, all thoracic functional units were isolated and underwent the flexion of 7.50 N.m. Average IDPs were measured during IVD relaxation of FE simulations (Fig. 13) and compared with *in-vitro* test^63^. In the *in-vitro* test^63^, thirty fresh-frozen human functional spinal units from 19 donors with aligned spines, including at least one specimen per thoracic spinal segmental level, were prepared for experimental testing. Nineteen specimens were from female donors and 11 from male donors, while average donor age was 56 years, ranging from 43 to 75 years^63^. Intradiscal pressure measurement was performed using a sensor device with a diameter of 1.2 mm (FMSPEZ50, MIPM GmbH, Hattenhofen, Germany), including a piezoelectrical micro-pressure sensor^63^. Then, the specimens were loaded up to pure moments of 7.50 N.m^63^.

Results in Fig. 13 showed that maximum percentage difference between FE and *in-vitro* results for IDP was 11.76% which proved that IVD mechanical responses (IDPs) were close to *in-vitro* test^63^.

Both ligament and IVD mechanical responses highlighted here indicate that the internal mechanical responses of the shared datasets were closely aligned with the real mechanics of soft tissues tested in various *in-vivo* and *in-vitro* experiments. Both mechanical and ROM validations demonstrated high confidence in the models’ predictions. Moreover, mesh quality of mean, 42, and 16807 FE models were assessed and validated in the following sections.

#### Assessment of mean hexahedral mesh quality

The quality of the morphed mean hexahedral vertebras and IVDs, achieved through the BCPD++ method, was assessed and presented in Table 5. Among the mesh quality parameters, the Jacobian ratio was a commonly used measure in FE analyses, which evaluated the accuracy and reliability of numerical simulations^64–66^. A perfect finite element was defined in a reference system (ξ_1_, ξ_2_, ξ_3_), where each point in the reference element was associated with its corresponding point in the modelled domain (x_1_, x_2_, x_3_) through a mapping function F. The Jacobian matrix of the mapping function F, evaluated at a specific reference point ξ, was defined as $\frac{\partial F}{\partial {\rm{\xi }}}\left({\rm{\xi }}\right)$^67^. If the Jacobian was negative, the corresponding element could not be used for FE analysis. According to Oñate, Jacobian ratios between 0.30 and 0.80 were generally considered acceptable for most applications^68^.

**Table 5:** Mean hexahedral mesh qualification for IVDs and vertebras.

	Mesh Error	Mesh Warnings	Jacobian ratio	Aspect Ratio > 10	Max Angle on Quad Faces > 160
**Vertebras**					
**Min**.	0%	1.26%	0.40	0.14%	0.25%
**Max**.	0%	8.15%	0.80	0.93%	0.47%
**IVDs**					
**Min**.	0%	0.15%	0.60	0.58%	0.59%
**Max**.	0%	8.63%	0.90	4.78%	1.03%
**CEPs**					
**Min**.	0%	2.65%	0.70	2.15%	3.56%
**Max**.	0%	12.23%	0.90	7.76%	8.45%

#### Assessment and validation of mesh quality for virtual and patient-personalised FE models

To ensure that all elements in the deformed spine mesh (virtual and patient-personalised FE models) were valid for FE simulations, a post-processing step was performed on the mesh. Since the shape and elements of the spine might be distorted by the shape deformation applied through the SSM, it was important to evaluate the quality of the mesh. Specifically, the Jacobian of each hexahedral element was computed, and if it was found to be invalid (e.g., negative), further refinement was applied to validate the hexahedral mesh quality. For this purpose, the Sum-of-Squares (SOS) relaxation algorithm was employed to optimize the hexahedral meshes^69^. The available Matlab code from the GitHub repository^70^ was used, and it required the MOSEK 9.3 Matlab optimization solver. In this study, 16807 virtual and 42 patient-personalised FE models were subjected for this mesh quality evaluation process. Remarkably, the percentage of invalid elements was 0%.

## Usage Notes

**Fig. 14:**
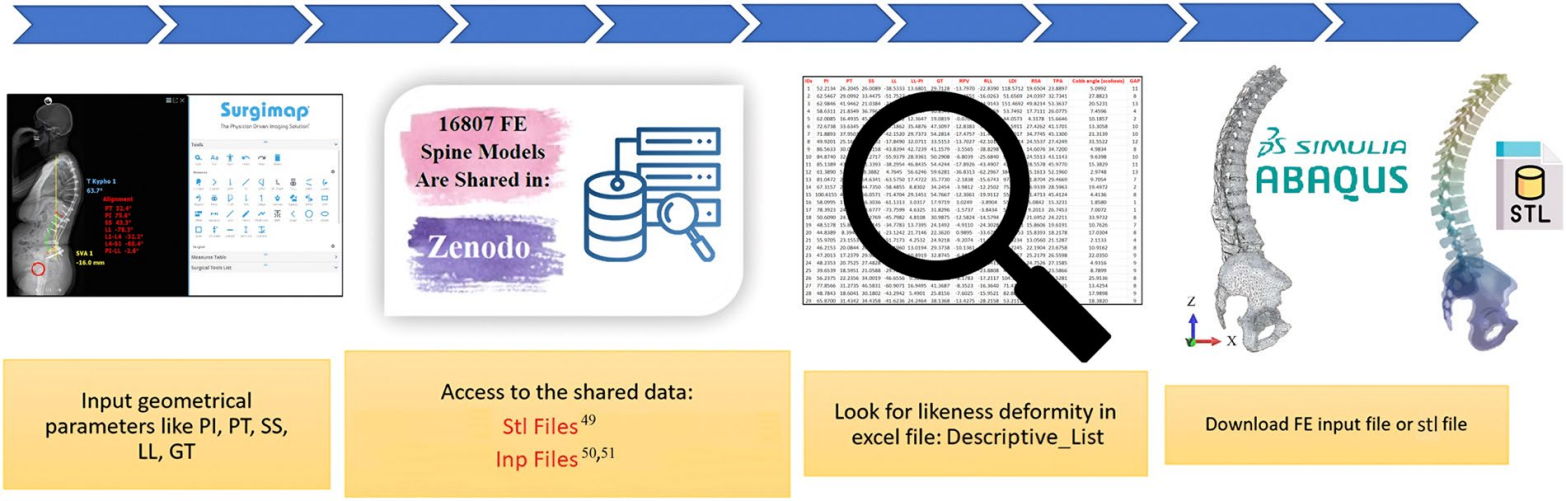
General usage guide for the shared data.

Clinicians and biomechanical researchers could utilize the provided excel file “Descriptive_List.xls” to search for a specific model of interest within the thoracolumbar spine morphology. Once identified, they could download the corresponding stl files and FE point coordinates. To generate FE input file for each of 16807 virtual models, the downloaded FE point coordinates could be substituted with the point coordinates in the mean FE input file “Mean_Model (Template).inp”. A comprehensive usage guide for the shared data was presented in Fig. 14.

The stl files could be opened using various image viewers, 3D modelling, and CAD programs such as Microsoft 3D Viewer (for Windows) and MeshLab (available on multiple platforms). Besides, an online open-source platform under an A-GPL3 license has been developed to facilitate access to data, in which the data can be filtered, visualized, and downloaded through the data visualization platform^71^.

Furthermore, the FE inp files could be opened using Abaqus 2019 and later versions. Other FE software that supported the inp extension could also open FE input files.

### Supplementary information

Supplementary Materials

## Data Availability

The BCPD++ open-source codes could be accessed at the GitHub repository^36^, and the SOS relaxation algorithm to check and repair the hexahedral meshes were available at the GitHub repository^70^. The repository for the online platform^71^ can be accessed at Github repository^72^.
