# Supplementary material for: Dataset of Finite Element Models of Normal and Deformed Thoracolumbar Spine
Source: Sci Data. 2024 May 29;11:549. doi: 10.1038/s41597-024-03351-8 (PMC11137096; doi:10.1038/s41597-024-03351-8)
Supplement: Supplementary file 1 — Supplementary Materials [file 41597_2024_3351_MOESM1_ESM.docx]

**Supplementary Materials:**

***Virtual cohort analyses***

Here, we examine the distribution of measured spinopelvic parameters among the 16807 virtual models, both triangulated and hexahedral. Recently Yilgor et al., 2017 demonstrated that integrated PI-proportional parameters can be utilized to measure sagittal deviation from a balanced spine, introducing the GAP score^S1^. The GAP score combines five subgroups: Relative Pelvic Version (RPV), Relative Lumbar Lordosis (RLL), Lumbar Distribution Index (LDI), Relative Spinopelvic Alignment (RSA), and an age factor^S1^. GAP ranges from 0 to 13, where 0 represents a balanced spine, and 13 represents the most severe sagittal deformity^S1^. We evaluate the GAP score for the virtual cohort and report the variances of geometrical spinopelvic parameters for each GAP value (ranging from 0 to 13) in Table S-1. Threshold analyses in Table S-1, conducted by the literature^S1^ further demonstrated that GAP values ≤2, 2< GAP ≤6, and GAP >6 correspond to proportioned (indicated by green values), moderately disproportioned (indicated by yellow values), and severely disproportioned (indicated by red values) spinal deformities, respectively.

Table S-1: Variances of spinopelvic parameters, and number of models in each GAP values (proportioned: green values, moderately disproportioned: yellow values, severely disproportioned: red values)

| ***GAP*** | ***Number of models*** | ***PI range (º)*** | ***SS range (º)*** | ***LL range (º)*** | ***LL-PI average (º)*** | ***GT range (º)*** | ***TPA average (º)*** |
| --- | --- | --- | --- | --- | --- | --- | --- |
| 1 | 653 | 34 to 87 | 33 to 63 | -80 to -40 | 6.15 | 7 to 36 | 17.56 |
| 2 | 1613 | 37 to 92 | 30 to 63 | -81 to -39 | 7.71 | -7 to 45 | 24.56 |
| 3 | 344 | 32 to 90 | 28 to 60 | -80 to -31 | 6.20 | 8 to 46 | 25.61 |
| 4 | 1154 | 30 to 91 | 26 to 58 | -80 to -25 | 7.47 | -7 to 45 | 22.80 |
| 5 | 1164 | 30 to 92 | 22 to 67 | -80 to -14 | 13.91 | -9 to 45 | 31.57 |
| 6 | 664 | 31 to 92 | 27 to 65 | -81 to -26 | 10.14 | 5 to 52 | 24.52 |
| 7 | 864 | 30 to 94 | 21 to 59 | -80 to -19 | 8.06 | 10 to 50 | 18.39 |
| 8 | 1082 | 32 to 95 | 21 to 55 | -70 to -15 | 16.10 | -8 to 54 | 29.62 |
| 9 | 936 | 31 to 94 | 20 to 55 | -68 to -11 | 21.13 | 18 to 57 | 33.48 |
| 10 | 2116 | 32 to 98 | 4 to 53 | -65 to 19 | 34.36 | 17 to 84 | 46.77 |
| 11 | 1058 | 31 to 99 | 6 to 51 | -61 to 16 | 28.57 | 19 to 84 | 40.81 |
| 12 | 2041 | 32 to 101 | 14 to 49 | -57 to -2 | 27.66 | 19 to 82 | 34.71 |
| 13 | 3118 | 35 to 101 | 5 to 38 | -50 to 0 | 40.22 | 27 to 76 | 57.91 |

The data presented in Table S-1 provide insights into the characteristics of the spine deformities based on the GAP score. For a balanced or proportioned balanced spine (GAP score of 1), the pelvic incidence (PI) ranges from 34º to 87º, which aligns with findings from previous research^S2^. Additionally, the results demonstrate a positive correlation between the GAP score and PI (Pearson correlation coefficient of 0.60).

Further analysis of the data in Table S-1 reveals a positive correlation between the GAP score and lumbar lordosis (LL) (Pearson correlation coefficient of 0.76). It is noteworthy that the average LL values for proportioned, moderately disproportioned, and severely disproportioned GAP scores are -65.58º, -60.35º, and -34.36º, respectively. This observation aligns with previous research^S3^, suggests that severe sagittal deformities can lead to a decrease in LL. A reduction in LL can result in various issues such as low back pain, intervertebral disc degeneration, and reduced mobility. To compensate for the loss of LL, the body naturally adopts compensation mechanisms by altering the posture and alignment of other parts of the spine. One such mechanism is pelvic retroversion, which involves the backward rotation of the pelvis. This retroversion helps to compensate for the loss of LL and maintain a more upright posture. The research^S4^ demonstrated that pelvic retroversion, characterized by the backward rotation of the pelvis, can decrease the sacral slope (SS) angle. Vrtovec et al., 2012 also established that the normal or ideal SS value typically falls within the range of 30º to 40º^S5^. The data analysis presented in Table S-1 further confirms these findings, as the average SS values decrease significantly (from 47º to 32º) as the GAP score progresses from proportioned to severely disproportioned. This reduction in SS values indicates that pelvic retroversion plays a role in aligning the SS values closer to the ideal range. In summary, the development of spine deformities or an increase in the GAP score leads to a decrease in lumbar lordosis (LL). However, as a compensation mechanism, pelvic retroversion stabilizes body posture by decreasing the sacral slope (SS) values. This interplay between LL, SS, and the GAP score highlights the complex relationships within the spine and the compensatory mechanisms that come into play as deformities progress.

Another factor of spinal imbalance is the mismatch between lumbar lordosis (LL) and pelvic incidence (PI)^S6^. When there is a discrepancy between LL and PI, the spine may compensate by altering the curvature in other regions, such as pelvic retroversion, as described earlier. Results in Table S-1 showed that the average LL-PI values for proportioned, moderately disproportioned, and severely disproportioned GAP scores are 6.93º, 9.43º, and 25.16º, respectively. This indicates that severe disproportioned GAP scores (ranging from 7 to 13) exhibit the highest LL and PI mismatching. There is no universally agreed-upon threshold for the degree of mismatch between PI and LL that is considered clinically significant. However, some studies have suggested that a PI-LL difference greater than 10º may be associated with an increased risk of spinal pathologies, such as spondylolisthesis or spinal stenosis^S7^.

Further analysis of the data in Table S-1 reveals a correlation between the GAP score and global tilt (GT) (Pearson correlation coefficient of 0.51). This correlation confirms that the retroversion of the sacral slope (RSA), one of the components of the GAP score, is dependent on GT. Additionally, the average GT values for proportioned, moderately disproportioned, and severely disproportioned GAP scores are 23.75º, 25.80º, and 38.78º, respectively. An increase in GT corresponds to a decrease in the anterior tilt of the pelvis and an increase in the posterior tilt of the pelvis. Therefore, as the GAP score increases, there is a decrease in LL, a backward tilt of the pelvis, and an increase in GT. This increase in GT is consistent with the notion that the spine column falls downward due to the decrease in LL^S8^.

The T1 pelvic angle (TPA) is a measurement used to evaluate the sagittal balance of the spine, specifically the relationship between the thoracic and pelvic regions^S9^. Analyzing the data in Table S-1, it is observed that the average TPA values for proportioned, moderately disproportioned, and severely disproportioned GAP scores are 21.06º, 26.12º, and 37.38º, respectively. This confirms that the development of spine deformities leads to an increase in the TPA angle, in line with previous findings^S10^.

Interestingly, 2266, 3326, and 11215 models are shared as examples of balanced, moderately misaligned, and severely misaligned spines, respectively (Table S-1). The box-and-whisker distribution of the GAP subgroups (RPV, RLL, LDI, and RSA) is presented in Fig. S-1.

Fig. S-1: Box-and-whisker distribution of GAP subgroups (RPV, RLL, LDI, and RSA)

The results presented in Fig. S-1 demonstrate the median values for RPV, RLL, LDI, and RSA as -10.77º, -25.15º, 70.23º, and 21.81º, respectively. Additionally, Fig. S-1 depicts the range from the first quartile (Q1) to the third quartile (Q3), as well as the minimum and maximum values for each of the GAP subgroups. The Q1 to Q3 range measures the spread of the middle 50% of the dataset. Specifically, the Q1 to Q3 ranges for RPV, RLL, LDI, and RSA are -18º to -5º, -36º to -13º, 55º to 116º, and 13º to 30º, respectively. These findings indicate that a significant portion of the shared data (10419 models) exhibits moderate and severe retroversion in RPV, which aligns with the GAP subgroup scale^S1^. Further exploration reveals that models 11802, 13927, and 14317 exhibit moderate and severe hypolordosis in RLL, aligned and hyperlordotic maldistribution in LDI, and moderate and severe positive malalignment in RSA, respectively. The presence of a high number of moderate and severe RSA models confirms that a considerable portion of the shared data demonstrates misaligned GT. Similarly, the abundance of moderate and severe hypolordosis RLL models supports the notion that a substantial portion of the shared data exhibits severe GAP scores and reduced LL, as indicated in Table S-1. Among the GAP components, LDI has the highest number (6334) of aligned models. Further analysis reveals strong correlations between GAP and RPV, RLL, LDI, and RSA (Pearson correlation coefficients of 0.80, 0.84, 0.81, and 0.77, respectively). It is worth noting that Yilgor et al., 2017^S1^ did not establish upper and lower bounds for GAP component variations, but the virtual dataset here provides insights into these variations, as depicted in Fig. S-1. The distribution of individual spinopelvic parameters (PI, PT, SS, LL, GT, and TPA) is illustrated in Fig. S-2.

Fig. S-2: Box-and-whisker distribution of individual spinopelvic parameters (PI, PT, SS, LL, GT, and TPA)

The median values for individual PI, PT, SS, LL, GT, and TPA are 58º, 25º, 33º, -40º, 35º, and 30º, respectively, as shown in Fig. S-2. The Q1 to Q3 ranges for PI, PT, SS, LL, GT, and TPA are 46º to 72º, 16º to 36º, 24º to 40º, -56º to -23º, 23º to 46º, and 19º to 38º, respectively. These findings indicate that most of the shared data exhibits high PT values (within the balanced PT range of 0º to 10º^S11^). Additionally, the PI variations between 46º and 72º (Q1 to Q3 range) correspond to an ideal LL range of -63º to -78º (calculated using the formula^S12^: ideal LL = ((PI × 0.54 + 12.70) × 1.08 + 21.61)). Consequently, most of the models demonstrate low LL values (LL Q1 to Q3 range: -56º to -23º), as mentioned earlier. Moreover, the ideal GT for the interquartile range is 7º to 19º (calculated using the formula^S1^: ideal GT = PI × 0.48 -15). Therefore, most of the models exhibit high GT values (GT Q1 to Q3 range: 23º to 46º), consistent with the prior information.

Furthermore, the scoliosis Cobb angle was assessed for both real and virtual data. The results indicated that there is no correlation between GAP and the scoliosis Cobb angle (Pearson correlation coefficient of -0.02). Generally, surgical treatment for scoliosis is indicated when the Cobb angle exceeds 45º^S13^. Interestingly, among the virtual cases, none had a Cobb angle greater than 45º, and approximately 5000 cases had a Cobb angle less than 10º. It is worth noting that four categories can be defined based on the combination of scoliosis and sagittal deformity:

1. No scoliotic (Cobb angle < 10º) and balanced sagittal alignment (GAP 1,2): 430 models.
2. No scoliotic (Cobb angle < 10º) and deformed sagittal alignment (GAP > 2): 4527 models.
3. Scoliosis (Cobb angle > 10º) and balanced sagittal alignment (GAP 1,2): 1836 models.
4. Scoliosis (Cobb angle > 10º) and deformed sagittal alignment (GAP > 2): 10,014 models.

Furthermore, IVD thickness was assessed for the shared dataset. IVD thickness assessment revealed that there is no significant correlation between IVD thickness and GAP (Pearson correlation coefficient < 0.50). This finding indicates that the GAP score does not incorporate information about IVD thickness. IVD thickness can be valuable for researchers seeking to incorporate disc thickness considerations into their analyses or treatment planning processes.

**References:**

[S1] Yilgor, C., Sogunmez, N., Boissiere, L., Yavuz, Y., Obeid, I., Kleinstück, F., Pérez-Grueso, F. J. S., Acaroglu, E., Haddad, S., Mannion, A. F., Pellise, F., & Alanay, A. Global Alignment and Proportion (GAP) Score. Journal of Bone and Joint Surgery, 99(19), 1661–1672 (2017).

[S2] Chen, H.-F., & Zhao, C.-Q. Pelvic incidence variation among individuals: functional influence versus genetic determinism. Journal of Orthopaedic Surgery and Research, 13(1), 59 (2018).

[S3] Silva, F. E., & Adams, M. A. The Spine chapter: Fundamentals of Biomechanics: Equilibrium, Motion, and Deformation. Springer International Publishing, 231–276 (2019).

[S4] Legaye, J., Duval-Beaupre, G., Marty, C., & Hecquet, J. Pelvic incidence: a fundamental pelvic parameter for three-dimensional regulation of spinal sagittal curves. European Spine Journal, 7(2) (1998).

[S5] Vrtovec, T., Janssen, M. M. A., Likar, B., Castelein, R. M., Viergever, M. A., & Pernuš, F. A review of methods for evaluating the quantitative parameters of sagittal pelvic alignment. The Spine Journal, 12(5), 433–446 (2012).

[S6] Mac-Thiong, J.-M., Roussouly, P., Berthonnaud, É., & Guigui, P. Sagittal Parameters of Global Spinal Balance. Spine, 35(22), E1193–E1198 (2010).

[S7] Boulay, C., Tardieu, C., Hecquet, J., Benaim, C., Mouilleseaux, B., Marty, C., Prat-Pradal, D., Legaye, J., Duval-Beaupère, G., & Pélissier, J. Sagittal alignment of spine and pelvis regulated by pelvic incidence: standard values and prediction of lordosis. European Spine Journal, 15(4), 415–422 (2006).

[S8] le Huec, J. C., Thompson, W., Mohsinaly, Y., Barrey, C., & Faundez, A. Sagittal balance of the spine. European Spine Journal, 28(9), 1889–1905 (2019).

[S9] Roussouly, P., & Nnadi, C. Sagittal plane deformity: an overview of interpretation and management. European Spine Journal, 19(11), 1824–1836 (2010).

[S10] Ryan, D. J., Protopsaltis, T. S., Ames, C. P., Hostin, R., Klineberg, E., Mundis, G. M., Obeid, I., Kebaish, K., Smith, J. S., Boachie-Adjei, O., Burton, D. C., Hart, R. A., Gupta, M., Schwab, F. J., & Lafage, V. T1 Pelvic Angle (TPA) Effectively Evaluates Sagittal Deformity and Assesses Radiographical Surgical Outcomes Longitudinally. Spine, 39(15), 1203–1210 (2014).

[S11] Czaprowski, D., Stoliński, L., Tyrakowski, M., Kędra, A., & Kotwicki, T. Non-invasive evaluation of posterior pelvic tilt using a skin-surface device: a reliability study in healthy individuals. Scoliosis Spinal Disord, 11(5) (2016).

[S12] Legaye, J., & Duval-Beaupère, G. Sagittal plane alignment of the spine and gravity: a radiological and clinical evaluation. Acta Orthopaedica Belgica, 71(2), 213–220 (2005).

[S13] Maruyama, T., & Takeshita, K. Surgery for Idiopathic Scoliosis: Currently Applied Techniques. Clinical Medicine. Pediatrics, 3 (2009).
